# USP15 regulates p66Shc stability associated with Drp1 activation in liver ischemia/reperfusion

**DOI:** 10.1038/s41419-022-05277-8

**Published:** 2022-09-26

**Authors:** Xinyao Tian, Yan Zhao, Zhe Yang, Qianrang Lu, Lin Zhou, Shusen Zheng

**Affiliations:** 1 Division of Hepatobiliary and Pancreatic Surgery, Department of Surgery, The First Affiliated Hospital, Zhejiang University School of Medicine, Hangzhou, China; 2 Key Laboratory of Organ Transplantation, Research Center for Diagnosis and Treatment of Hepatobiliary Diseases, Hangzhou, China; 3 Department of Pharmacology, Dalian Medical University, Dalian, China; 4 Department of Hepatobiliary and Pancreatic Surgery, Department of Liver Transplantation, Shulan (Hangzhou) Hospital, Hangzhou, China

**Keywords:** Ubiquitylation, Apoptosis, Deubiquitylating enzymes, Liver diseases

## Abstract

Liver ischemia/reperfusion (I/R) injury is a major clinical concern of liver transplantation, which accounts for organ rejection and liver dysfunction. The adaptor protein p66Shc acts as a crucial redox enzyme and is implicated in liver I/R. Elevated p66Shc expression is associated with hepatocellular apoptosis in liver I/R, but the molecular mechanisms of p66Shc responsible for its aberrant expression and function remain unknown. In the present study, hepatocyte-specific p66Shc-knockdown mice exhibited clear inhibition in hepatocellular apoptosis and oxidative stress under liver I/R, while hepatocyte-specific p66Shc overexpression mice displayed the deteriorative impairment. Mechanistically, p66Shc-triggered mitochondrial fission and apoptosis in liver I/R by mediating ROS-driven Drp1 activation. Furthermore, a screening for p66Shc-interacting proteins identified ubiquitin-specific protease 15 (USP15) as a mediator critical for abnormal p66Shc expression. Specifically, USP15 interacted with the SH2 domain of p66Shc and maintained its stabilization by removing ubiquitin. In vivo, p66Shc knockdown abrogated USP15-driven hepatocellular apoptosis, whereas p66Shc overexpression counteracted the antiapoptotic effect of USP15 silencing in response to liver I/R. There was clinical evidence for the positive association between p66Shc and USP15 in patients undergoing liver transplantation. In summary, p66Shc contributes to mitochondrial fission and apoptosis associated with Drp1 activation, and abnormal p66Shc expression relies on the activity of USP15 deubiquitination under liver I/R. The current study sheds new light on the molecular mechanism of p66Shc, and identifies USP15 as a novel mediator of p66Shc to facilitate better therapeutics against liver I/R.

## Introduction

Liver ischemia/reperfusion (I/R) remains a major challenge triggered by liver surgery. Serious postoperative complications, such as liver dysfunction and organ failure, may ensue as a result of reperfusion injury, which remains a serious challenge during liver transplantation. Some therapeutic strategies for liver I/R have been reported, including ischemia preconditioning, pharmacological agents, surgical interventions and gene therapy [1]. Unfortunately, no specific therapies are currently available for liver I/R in clinical practice.

Liver I/R is characterized by hepatocellular apoptosis, which is mainly mediated by reperfusion-induced reactive oxygen species (ROS) production [2]. p66Shc, 66 kDa isoform of ShcA, emerges as a redox-sensitive enzyme that contributes to mitochondrial ROS production [3]. p66Shc expression is dramatically increased in liver injury, and inhibition of p66Shc expression and activity is sufficient to attenuate hepatocellular damage, following nonalcoholic liver disease (NAFLD)-, alcoholic liver disease (ALFD)-, or acetaminophen (APAP)-induced injury and liver fibrosis [4–7]. Notably, downregulation of p66Shc alleviates apoptosis and oxidative stress induced by liver I/R [8]. These results indicate that p66Shc could be a promising target of liver injury.

Hepatocyte apoptosis is initiated upon liver I/R injury. Emerging evidence points that mitochondrial dynamics is implicated in apoptosis during liver I/R. p66Shc is associated with disturbances in mitochondrial dynamics under the conditions of diabetic nephropathy and APAP-induced liver injury [6, 9], but the mechanisms of p66Shc remain to be further clarified. Mitochondria are highly dynamic organelles that constantly undergo fission and fusion to confer widespread benefits on mitochondrial function. Under high cellular stress, excessive mitochondrial fission can facilitate apoptosis [10], which is finely controlled by mitochondrial fission protein Drp1 [11, 12]. Recent research has revealed that inhibition of Drp1 activation could protect hepatocytes from mitochondrial fission and apoptosis in liver I/R [13, 14], and that mitochondrial ROS are involved in Drp1 activation [15]. It is likely that p66Shc, a redox-sensitive enzyme in mitochondria, contributes to Drp1/mitochondrial fission and apoptosis via ROS production in liver I/R.

p66Shc functions as a master regulator of ROS, leading to excessive cellular ROS production. Following phosphorylation at Ser36 by protein kinase C (PKC), p66Shc translocates to mitochondria and then oxidizes cytochrome c, resulting in mitochondrial hydrogen peroxide (H_2_O_2_) formation [16]. In addition, p66Shc also suppresses FOXO-dependent transcription of antioxidants [17]. In turn, oxidative stress could trigger p66Shc phosphorylation by PKC and p66Shc transcription following p53 activation [18–20]. Sirtuin 1 could deacetylate p66Shc, thus inhibiting p66Shc-mediated mitochondrial ROS generation [21, 22]. Considering the critical function of p66Shc in ROS production and liver injury, it is essential to explore novel factors responsible for regulating the expression and function of p66Shc.

The function of p66Shc is tightly associated with posttranslational modifications such as phosphorylation and deacetylation, et al. However, whether ubiquitination, the major route of protein degradation [23], is implicated in regulating p66Shc stability remains to be clarified. Deubiquitinases (DUBs) are crucial for protein expression through catalyzing the removal of ubiquitin from substrate proteins. USP15, a member of ubiquitin-specific protease, is exploited in diverse cellular functions through regulating specific substrates [24]. USP15 promotes the deubiquitination of MDM2 to inhibit cancer-cell apoptosis and T-cell responses [25]. USP15 functions in DNA-end resection by deubiquitinating BARD1 in breast cancer [26]. USP15 physically interacts with XOR and stabilizes Kelch-like ECH, which is attributed to ROS accumulation in hepatocellular carcinoma [27]. Using tandem affinity purification/mass spectrometry (TAP/MS) and co-immunoprecipitation (Co-IP)/MS, we identified the interaction between USP15 and p66Shc. Therefore, we speculate that USP15 may contribute to the increased expression of p66Shc during liver I/R.

This study aimed to investigate the molecular mechanism of p66Shc in liver I/R and characterize the interaction between USP15 and p66Shc. First, our data indicate that p66Shc contributes to mitochondrial fission and apoptosis associated with Drp1 activation. Then, p66Shc is specifically deubiquitinated by USP15. USP15 potentiates apoptosis and oxidative stress in liver I/R by stabilizing p66Shc expression. Our results shed new light on the enhancing effect of p66Shc and reveal a novel regulatory mechanism underlying p66Shc upregulation in liver I/R, which can be exploited for future treatment strategies.

## Materials and methods

### Clinical samples

Human liver samples were collected at Shulan Hospital (Hangzhou, China) from 2019.5.1 to 2020.12.20. Control liver samples were biopsied from the left lobe of donor livers during pre-orthotopic liver transplantation evaluation. Liver transplantation samples were intra-operatively biopsied from the left lobe of liver of transplant patients 2–3 h after portal reperfusion (before abdominal closure). The patients underwent liver transplantation owing to advanced liver cirrhosis or hepatocellular carcinoma. Written consent was obtained and the information of clinical samples was shown in Table S1. All procedures were approved by the Human Ethics Committees of Shulan Hospital (Hangzhou) and were conducted in accordance with the principles of the Declaration of Helsinki.

### Animals

As described, 70% partial warm liver I/R model was generated in mice [28]. Briefly, C57BL/6 mice (8–10-week-old, male, 18–22 g) were provided a normal laboratory chow diet and water. Mice were grouped randomly (10–12 mice each group). The artery and portal veins of mice were interrupted for 1 h, and then blood supply was restored for the indicated time points. After 6 h of reperfusion, plasma and liver tissue were harvested for further analysis. Mice in the sham group were conducted with the same procedure without interruption of the hepatic blood supply. Adeno-associated virus (AAV) and adenovirus (AV) were obtained from Hanbio Biotechnology (Shanghai, China). Mice were administered a single intravenous injection of AAV three weeks or AV one week before liver I/R. The shRNA sequences (Hanbio Biotechnology) were listed in Table 1. All experimental procedures were approved by the Ethics Committee of Dalian Medical University (Dalian, China) and were conducted in accordance with the Care and Use of Laboratory Animal Guides.

**Table 1:** siRNA and shRNA sequences of genes.

Gene	5’-3’
p66Shc siRNA	GCUGCAUCCCAACGACAAATT
USP15 siRNA	CCAAAGAUCUCUCCUUCAUTT
Negative control	UUCUCCGAACGUGUCACGUTT
p66Shc shRNA	AAGGTATATTGCTGTTGACAGTGAGCGGCTGCATCCCAACGACAAATAGTGAAGCCACAGATGTATTTGTCGTTGGGATGCAGCTGCCTACTGCCTCG
USP15 shRNA	AAGGTATATTGCTGTTGACAGTGAGCGGCACTGAAGAAACATGCAATAGTGAAGCCACAGATGTATTGCATGTTTCTTCAGTGCTGCCTACTGCCTCG

### Biochemical analysis

The indicators of hepatocellular injury, including serum alanine aminotransferase (ALT), aspartate aminotransferase (AST) and lactic dehydrogenase (LDH) levels were measured using commercial kits (Jiancheng, Nanjing, China).

The indicators of oxidative stress, including catalase (CAT), H_2_O_2_, malondialdehyde (MDA) and superoxide dismutase (SOD) levels were detected using commercial kits (Jiancheng).

### Histology and immunohistochemistry staining

Liver specimens were fixed and stained with hematoxylin and eosin (H&E). Immunohistochemistry (IHC) for p66Shc was conducted as follows: liver paraffin sections were incubated with antibody against p66Shc (BD Biosciences, USA) and then were stained with DAB and hematoxylin.

### Cell transfection and treatment

AML12 cells (ATCC, Manassas, VA, USA) were cultured in DMEM: F12 medium (Gibco, Carlsbad, CA, USA) supplemented with FBS (Gibco), ITTS, and dexamethasone. AML12 cells were exposed to hypoxia/reoxygenation (H/R) as described previously [29]. In brief, AML12 cells were cultured at 1% O_2_ for 12 h, and then at 21% O_2_ conditions for the indicated time points. Plasmids or small interfering RNAs (siRNAs) were transfected to AML12 cells using lipofectamine 3000 (Invitrogen, Carlsbad, CA, USA) for 48 h before H/R. The specific siRNA sequences (GenePharma, Suzhou, China) were listed in Table 1. After transfection, AML12 cells were incubated with 100 μM MitoTEMPO (Sigma, St Louis, Missouri, USA) or 10 μM Mdivi-1 (Selleck, Shanghai, China) for 3 h, followed by H/R. In addition, 20 μM MG132 (Selleck, USA) was administered for 3 h or 100 μM cycloheximide (CHX, Sigma) was applied for 0, 1, 2, and 4 h to AML12 cells after USP15 siRNA transfection.

HEK293 cells were grown in DMEM (Gibco) medium containing FBS (Gibco), penicillin and streptomycin (Beyotime, Hangzhou, China). Relevant plasmids including Myc-p66Shc, Flag-USP15 WT, Flag-USP15 C269A, and HA-Ub (GenePharma) were transfected into HEK293 cells using lipofectamine 3000 for 24–72 h, followed by a Co-IP assay.

### RNA isolation and quantitative real-time PCR

After extracted using TRIzol (Invitrogen), total RNA was synthesized to cDNAs using *Evo M-MLV* RT kit (Accurate Biotechnology, Changsha, China). qRT-PCR was carried out using SYBR Green Premix *Pro Taq* HS qPCR Kit (Accurate Biotechnology). Gene expressions were normalized to β-actin. PCR primers (Sangon Biotech, Shanghai, China) were shown in Table 2.

**Table 2:** Primer sequences of genes.

Gene	Forward primer (5′–3′)	Reverse primer (5′–3′)
β-actin (mice)	GTGCTATGTTGCTCTAGACTTCG	ATGCCACAGGATTCCATACC
IL-1β (mice)	TCGCAGCAGCACATCAACAAGAG	AGGTCCACGGGAAAGACACAGG
IL-6 (mice)	CTCCCAACAGACCTGTCTATAC	CCATTGCACAACTCTTTTCTCA
p66Shc (mice)	ACTACCCTGTGTTCCTTCTTTC	TCGGTGGATTCCTGAGATACTGT
TNF-α (mice)	ATGTCTCAGCCTCTTCTCATTC	GCTTGTCACTCGAATTTTGAGA

### Western blotting

Proteins extracted from liver tissues and AML12 cells were subjected to SDS-PAGE. The target antigens were probed using specific antibodies overnight at 4 °C. The following antibodies were used: anti-p66Shc (610878, BD Biosciences); anti-USP15 (14354-1-AP, Proteintech Group, Wuhan, China), anti-USP9X (55054-1-APProteintech), anti-Drp1 (12957-1-AP, Proteintech), anti-HA (66006-2-Ig, Proteintech), anti-Flag (66008-3-Ig, Proteintech), anti-Myc (60003-3-Ig, Proteintech), anti-phosphory-Drp1^Ser616^ (3455, Cell Signaling Technology, Danvers, MA, USA), anti-phosphory-Drp1^Ser637^ (4867, Cell Signaling Technology), anti-Ubiquitin (3936, Cell Signaling Technology), anti-B-cell leukemia/lymphoma (Bcl2, A11313, ABclonal Biotechnology, Wuhan, China), anti-Bax (A15646, ABclonal), anti-cleaved caspase-3 (C-cas-3, abs132005, absin, Shanghai, China) and anti-β-actin (A5092, bimake, Shanghai, China). After HRP-conjugated secondary antibodies (Beyotime) incubation, the protein in bands were visualized using BeyoECL Plus (Beyotime). Uncropped Immunoblots are shown in Supplemental Figures WB.

### Co-IP/MS

The specific antibodies were incubated with Protein A/G magnetic beads (bimake) at room temperature for 4 h. Then, the extracted proteins were added into the mixtures of antibodies and beads at room temperature for 1 h. The bead-bound proteins were subjected to western blotting or MS.

For MS, the bead-bound proteins were separated by SDS-PAGE and visualized by Coomassie Brilliant Blue staining. Then the gels were digested, and the peptides were obtained from the gels. The anti-p66Shc IP-specific peptides were separated by an Easy nLC 1200 chromatographic system (Thermo Scientific) and then analyzed on a Q-Exactive HF-X mass spectrometer (Thermo Scientific). MaxQuant 1.6.1.0 was used to analyze the LC-MS/MS data. Peptide-spectrum match (PSM) false discovery rate (FDR) ≤ 0.01 and Protein FDR ≤ 0.01 were used to identify the peptide and protein, respectively.

### MitoSOX and MitoTracker staining

For mitochondrial ROS detection, AML12 cells were treated with 5 μM MitoSOX Red (Invitrogen) for 10–15 min along with Hoechst 33342 staining for 15 min in the dark. 200 nM MitoTracker Red CMXRos (Invitrogen) was employed for 10 min to visualize mitochondrial fragments. For the morphological analysis of mitochondria, a mitochondrion with a length less than 1 μm was regarded as fragmented [9, 30].

### TUNEL staining

Apoptosis induced by H/R was measured using In Situ Fluorescein TUNEL Cell Apoptosis Detection Kit (TransGen, Beijing, China). Apoptotic cells were labeled with terminal deoxynucleotidyl transferase (TdT) for 45–60 min at 37 °C. DAPI (Beyotime) was applied to stain the nuclei.

### JC-1 staining

Mitochondrial membrane potential (Δψm) was measured by JC-1 probe (Beyotime). Red fluorescent aggregates of JC-1 were located in normal mitochondria, while green fluorescent monomers of JC-1 transformed into depolarized mitochondria. The samples were treated with the JC-1 probe and stained with DAPI. The ratio of red to green JC-1 fluorescence was used to analyze Δψm alternation.

### Adenosine triphosphate (ATP) content

ATP content was measured following the protocol (Beyotime). Briefly, AML12 cells were incubated with ATP assay buffer and then the samples were collected by centrifugation. The supernatants were incubated with the ATP probe, and ATP content was measured using luminometer.

### Transmission electron microscopy (TEM)

Mitochondrial morphology was visualized using TEM. Liver tissue and AML12 cells were fixed in 2.5% glutaraldehyde followed by the incubation of osmium tetroxide. After washing with ethanol, the sections were stained immediately with lead citrate. Mitochondrial area and length were determined by ImageJ to quantify mitochondrial fragmentation [31, 32].

### Statistics

Data were presented as mean ± SD and were analyzed using GraphPad Prism software. Unpaired two-tailed Student’s *t* test was applied for two-group comparisons, and one-way ANOVA test was applied for multi-group comparisons. *P* values < 0.05 were considered to be a significant difference.

## Results

### p66Shc serves as an important driver of liver I/R

**Fig. 1:**
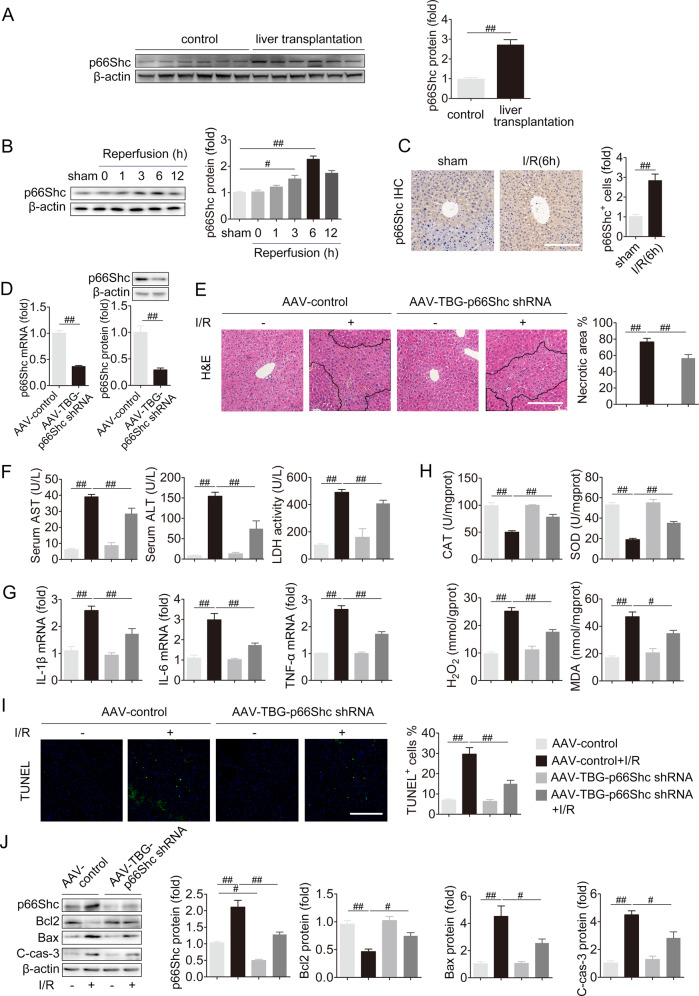
Hepatic-specific p66Shc knockdown protects against oxidative stress, apoptosis and inflammation in liver I/R. **A** The protein expression of p66Shc in the livers of normal human and patients undergoing liver transplantation, *n* = 6. **B** The protein expression of p66Shc in the livers of mice exposed to 1 h of ischemia followed by 0, 1, 3, 6, and 12 h of reperfusion, *n* = 3. **C** Immunohistochemistry staining for p66Shc in the livers of mice exposed to 1 h of ischemia followed by 6 h of reperfusion. Scale bar, 100 μm. **D**–**J** AAV-TBG-p66Shc shRNA was injected to mice followed by liver I/R. **D** p66Shc mRNA (*n* = 6) and p66Shc protein expression (*n* = 3). **E** H&E staining. Scale bar, 100 μm. **F** Serum ALT, AST and LDH levels, *n* = 8. **G** Liver IL-1β, IL-6 and TNF-α mRNA levels, *n* = 6. **H** Liver CAT, SOD, H_2_O_2_ and MDA contents, *n* = 8. **I** TUNEL staining. Scale bar, 100 μm. **J** p66Shc, Bcl2, Bax and, C cas-3 protein expression, *n* = 3. ^#^*P* < 0.05, ^##^*P* < 0.01.

First, we detected p66Shc expression in the livers of normal human and patients who had undergone liver transplantation to analyze the association of p66Shc with liver I/R. The p66Shc protein was remarkably increased challenged by liver I/R (Fig. 1A). Next, p66Shc expression was assessed in liver I/R models in vivo at various time points after reperfusion. Consistent with I/R injury in human, the protein expression of p66Shc was strikingly upregulated and reached a peak at 6 h of reperfusion in mice (Fig. 1B). Immunohistochemistry staining further confirmed the increased p66Shc expression in the livers of mice under I/R condition (Fig. 1C). These findings indicate that the elevated protein expression of p66Shc is related to liver I/R.

Then, we focused on the contribution of p66Shc to liver I/R. As expected, p66Shc knockdown by AAV-TBG-p66Shc shRNA in vivo exhibited clear reduction in hepatocellular necrosis after liver I/R (Fig. 1D, E). Additionally, the increased levels of hepatocellular damage markers (serum ALT, AST, and LDH) and inflammatory cytokines (IL-1β, IL-6, and TNF-α) in liver I/R were suppressed by p66Shc knockdown (Fig. 1F, G). More strikingly, compared to I/R stimulation, p66Shc silencing prevented the decreased activities of the antioxidant enzymes CAT and SOD, as well as the increased H_2_O_2_ and MDA contents (Fig. 1H). The number of apoptotic cells (TUNEL^+^) was significantly increased in mice with liver I/R, while p66Shc knockdown reversed this trend (Fig. 1I). Knockdown of hepatic p66Shc was accompanied by obvious increased Bcl2 levels and decreased Bax and C-cas-3 levels (Fig. 1J). The results in vitro were consistent with those in vivo. p66Shc siRNA protected AML12 cells from apoptosis in H/R (Fig. S1). Moreover, the protection of p66Shc knockdown on apoptosis after the peak of p66Shc expression during reperfusion was also maintained, as shown in Fig. S2. In contrast, the oxidative stress, inflammation, and apoptosis during liver injury were exacerbated by p66Shc overexpression both in vitro and in vivo (Fig. S3). Collectively, these findings reveal that p66Shc is pivotal for liver I/R-induced inflammation, oxidative stress, and apoptosis.

### p66Shc contributes to liver I/R by mediating mitochondrial fission and apoptosis associated with Drp1 activation

**Fig. 2:**
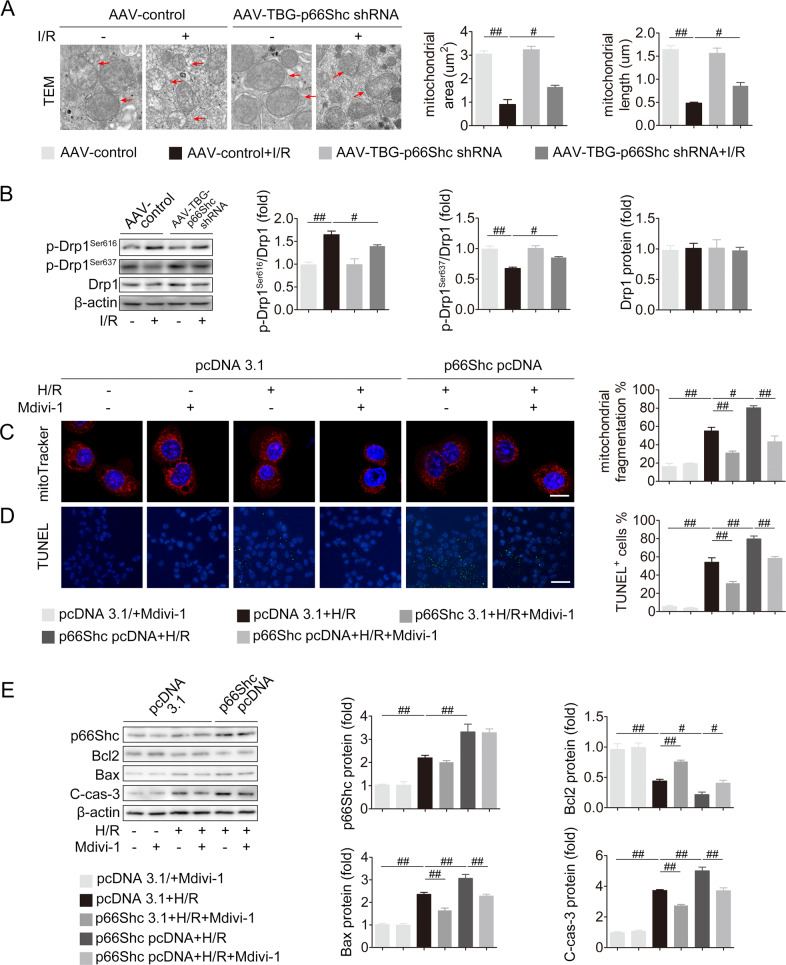
p66Shc contributes to liver I/R by mediating mitochondrial fission and apoptosis associated with Drp1 activation. **A**, **B** AAV-TBG-p66Shc shRNA was injected to mice followed by liver I/R. **A** Mitochondrial morphology measured using TEM (12000×, magnification). The red arrows indicate mitochondria. **B** p-Drp1Ser616, p-Drp1Ser637, and Drp1 protein expression. **C**–**F** AML12 cells were transfected with pcDNA-p66Shc and then stimulated with Mdivi-1 exposed to H/R. **C** Mitochondrial dynamics perturbation measured by MitoTracker (red). Scale bar, 12.5 μm. **D** TUNEL staining. Scale bar, 50 μm. **E** p66Shc, Bcl2, Bax, and C-cas-3 protein expression, *n* = 3. ^#^*P* < 0.05, ^##^*P* < 0.01.

We next gained insight into the underlying mechanism of p66Shc in liver I/R. p66Shc knockdown exhibited the protection against mitochondrial dysfunction in I/R or H/R, as evidenced by the decreased mitochondrial membrane potential, mitochondrial fragmentation and the increased ATP content (Fig. 2A and Fig. S4A–4D). The mitochondria displayed a swollen and small morphology during liver I/R or H/R, while p66Shc knockdown resulted in elongated mitochondria and less fragmentation (Fig. 2A and Fig. S4A). Interestingly, we found that silencing p66Shc inhibited the activation of Drp1 that emerges as a novel contributor to mitochondrial fission in liver I/R [13, 14], as revealed by the downregulation of p-Drp1^Ser616^ and upregulation of p-Drp1^Ser637^ under liver I/R or H/R conditions (Figs. 2B and S4E). Furthermore, p66Shc overexpression resulted in mitochondrial fission and apoptosis in AML12 cells exposed to H/R, which was blunted by Mdivi-1, a specific inhibitor of Drp1 (Fig. 2C–E). Hence, p66Shc-driven mitochondrial fission and cell apoptosis are associated with Drp1 activation in liver I/R. Accumulating evidence has revealed that Drp1 activation can be attributed to mitochondrial ROS accumulation [15, 33]. Upon H/R stimulation, scavenging mitochondrial ROS by MitoTEMPO resulted in the decrease of Drp1 activation, accompanied by the inhibition of mitochondrial fragmentation and cell apoptosis (Fig. S4F–4H). These results provide evidence that p66Shc-mediated Drp1 activation during liver I/R is attributed to mitochondrial ROS overproduction.

### USP15 interacts with p66Shc

**Fig. 3:**
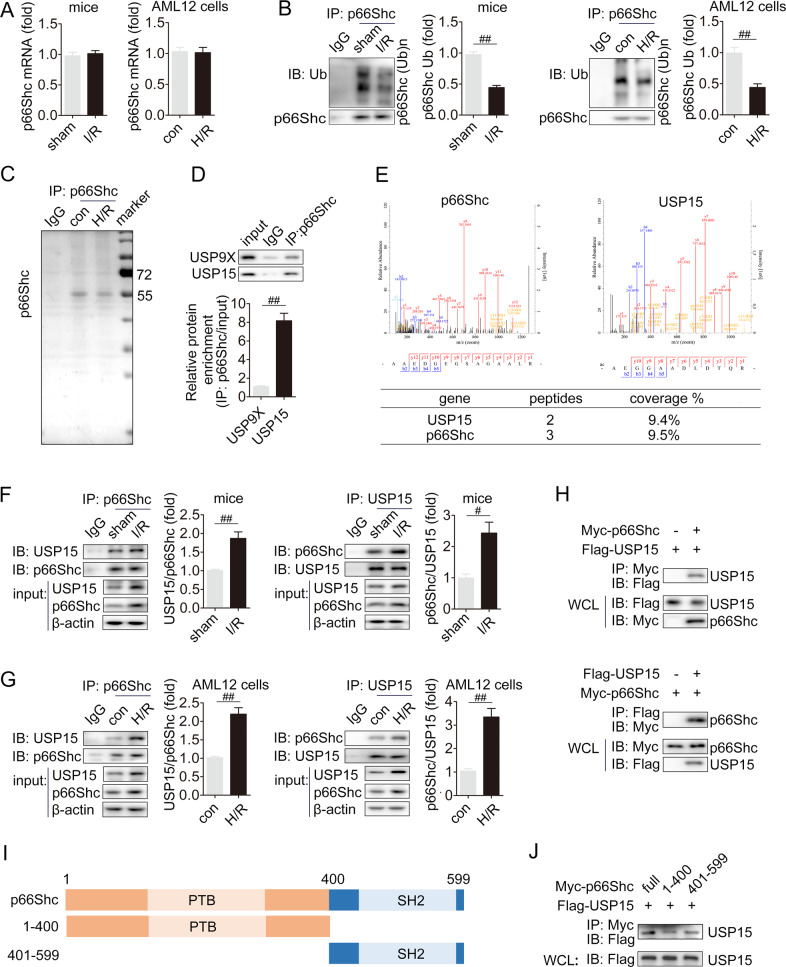
USP15 interacts with p66Shc. p66Shc mRNA (**A**) and p66Shc ubiquitination levels (**B**) in the livers of mice subjected to I/R and in AML12 cells exposed to H/R. **C** AML12 cells were exposed to H/R and then Co-IP/MS analysis was performed. Proteins interacting with p66Shc were separated and then visualized by Coomassie blue staining. **D** The interaction between endogenous p66Shc and USP15 or USP9X was detected in the livers of mice. **E** The interacting peptide between USP15 and p66Shc. **F**, **G** The interaction between endogenous p66Shc and USP15 was detected in the livers of mice exposed to I/R and in AML12 cells exposed to H/R. **H** HEK293 cells were transfected with Flag-USP15 and Myc-p66Shc as indicated. IP assay was conducted using anti-Flag or anti-Myc antibody. **I** Schematic representation of p66Shc and its mutants, showing that p66Shc protein contains a PTB domain and a SH2 domain (pfam: http://pfam.xfam.org/). **J** Flag-USP15 was co-transfected with Myc-p66Shc or either of its mutants, and their interaction was analyzed by IP. ^#^*P* < 0.05, ^##^*P* < 0.01.

We further evaluated the molecular mechanism underlying p66Shc upregulation in I/R injury. Although I/R exposure resulted in a substantial increase in p66Shc protein (Fig. 1B), p66Shc mRNA changed slightly in vivo and in vitro (Fig. 3A). Compared with the control group, p66Shc polyubiquitination levels were noticeably lower in the I/R and H/R groups (Fig. 3B). Thus, ubiquitylation may account for the increased stability of p66Shc, and we next sought to identify the DUB that is responsible for p66Shc instability in liver I/R. After IP with anti-p66Shc antibody in AML12 cells, proteins interacting with p66Shc were separated and then visualized by Coomassie blue staining (Fig. 3C). The following MS assay identified DUBs USP5, USP9X, USP10, USP14, USP15, USP43, USP47 and Dusp28 as potential p66Shc-interactors based on a research of Uniprot human database. In addition, TAP-MS has been performed to identify proteins interacting with p66Shc [34]. Among the proteins interacting with p66Shc, we were interested in DUBs, including USP9X, USP9Y, and USP15, as shown in BioGRID database (https://thebiogrid.org/) [35]. Then, we aimed to study the interactions of p66Shc with USP9X and USP15, two DUBs detected by both TAP/MS and Co-IP/MS. Co-IP assays detected that endogenous USP15, but not USP9X, strongly interacted with p66Shc (Fig. 3D). The following study focused on the interaction between USP15 and p66Shc, and the interacting peptides between them were shown in Fig. 3E. In both I/R and H/R, the protein expression of USP15 and p66Shc, as well as their interaction were increased (Fig. 3F, G). In addition, immunofluorescence staining showed that p66Shc (green) and USP15 (red) were colocalized in hepatocytes during H/R (Fig. S5). This indicated that either the upregulation of USP15 and p66Shc, or their increased interaction or both contribute to liver I/R. Furthermore, Myc-p66Shc and Flag-USP15 plasmids were co-transfected to HEK293 cells in order to verify the physical association between USP15 and p66Shc. As shown in Fig. 3H, USP15 and p66Shc efficiently interacted with each other. The truncated mutants of Myc-p66Shc were used to explore the fragments involved in the interaction (Fig. 3I). The SH2 domain of p66Shc was required and sufficient for binding to USP15 (Fig. 3J).

### USP15 promotes the deubiquitination of p66Shc

**Fig. 4:**
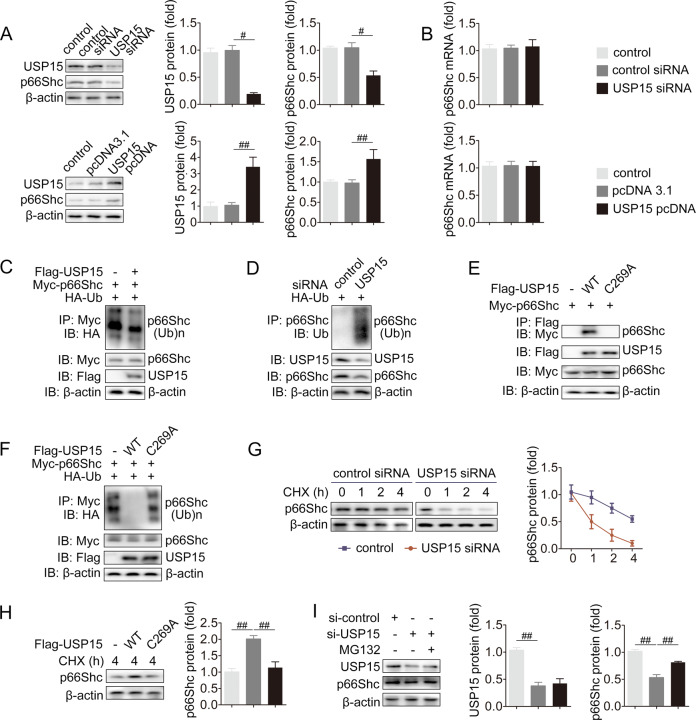
USP15 suppresses ubiquitination and degradation of p66Shc. USP15 siRNA or pcDNA-USP15 was transfected to AML12 cells. Then, p66Shc protein (**A**) and mRNA (**B**) were measured. **C** Flag-USP15, myc-p66Shc and HA-Ub were co-transfected into HEK293 cells as indicated. IP incubated with anti-HA antibody was performed to analyze p66Shc ubiquitination. **D** USP15 siRNA and HA-Ub were co-transfected into AML12 cells as indicated. IP with anti-HA antibody was performed to analyze the ubiquitination of p66Shc. **E** Myc-p66Shc and Flag-USP15 WT or C296A were co-transfected into HEK293 cells as indicated. IP incubated with anti-Flag antibody was performed to analyze the interaction between p66Shc and USP15. **F** Myc-p66Shc, Flag-USP15 WT or C269A and HA-Ub were co-transfected into HEK293 cells as indicated. IP with anti-HA antibody was performed to analyze the ubiquitination of p66Shc. **G** AML12 cells were transfected with USP15 siRNA followed by CHX treatment for the indicated periods. p66Shc expression was evaluated. **H** AML12 cells were transfected with Flag-USP15 WT or C296A followed by CHX treatment for 4 h. p66Shc expression was evaluated. **I** AML12 cells were transfected with USP15 siRNA followed by MG132 treatment. The protein expression of p66Shc was evaluated. ^#^*P* < 0.05, ^##^*P* < 0.01.

Considering the interaction between USP15 and p66Shc as well as the deubiquitinating activity of USP15, USP15 may exert an effect on p66Shc deubiquitination. p66Shc protein was remarkably decreased upon USP15 knockdown and increased upon USP15 overexpression (Fig. 4A). Nevertheless, neither USP15 knockdown nor USP15 overexpression resulted in apparent changes in p66Shc mRNA (Fig. 4B). Having determined the regulation of USP15 on p66Shc protein, we next investigated whether USP15 catalyzes p66Shc deubiquitination. HA-Ub, Myc-p66Shc, and Flag-USP15 plasmids were co-transfected to HEK-293 cells. Flag-USP15 significantly reduced p66Shc ubiquitin conjugation (Fig. 4C). In contrast, USP15 knockdown substantially increased endogenous p66Shc ubiquitination, accompanied by a decrease in p66Shc protein in AML12 cells (Fig. 4D). USP15 features two consensus sequences harboring a classical catalytic triad (Cys-His-Asp) [36]. It has been identified that the deubiquitinase activity of USP15 is dependent on Cys269 [37, 38]. In addition, a catalytically inactive mutant of USP15 (USP15 C269A) not only showed weaker binding with p66Shc but also increased the accumulation of p66Shc ubiquitination (Fig. 4E, F). Thus, the interaction between USP15 and p66Shc mainly depends on the deubiquitylating activity of USP15.

Our findings support the hypothesis that USP15 functions as a p66Shc DUB and mediates its protein stability. In fact, following CHX treatment, USP15 suppression significantly resulted in much faster degradation of p66Shc (Fig. 4G). USP15 overexpression prolonged the p66Shc protein half-life, whereas the catalytically inactive USP15 shortened it (Fig. 4H). Additionally, p66Shc degradation driven by USP15 siRNA was rescued by the proteasome-specific inhibitor MG132, indicating that p66Shc degradation was mediated by a proteasomal pathway (Fig. 4I). Therefore, USP15 suppresses the ubiquitination and degradation of p66Shc.

### USP15 is required for oxidative stress, apoptosis, and inflammation in liver I/R

**Fig. 5:**
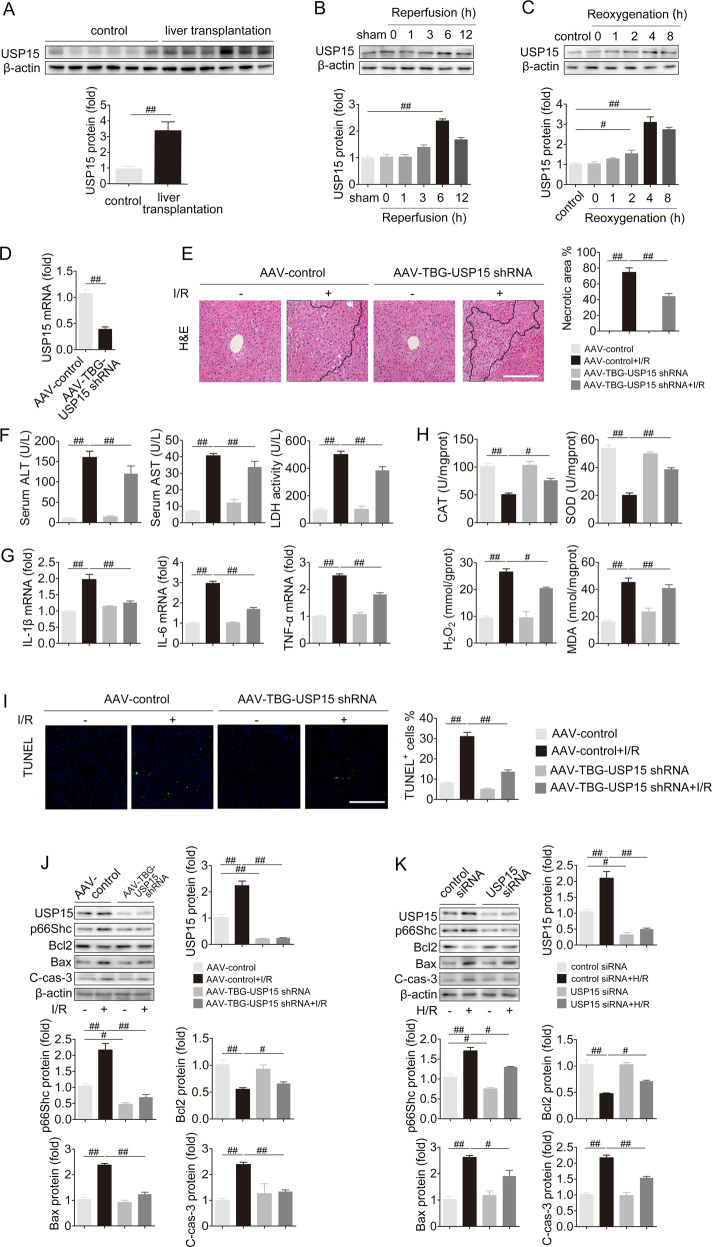
USP15 knockdown protects against oxidative stress, apoptosis, and inflammation in liver I/R. **A** The protein expression of USP15 in the livers of normal human and patients undergoing liver transplantation, *n* = 6. (B) USP15 protein expression in the livers of mice exposed to 1 h of ischemia followed by 0, 1, 3, 6, and 12 h of reperfusion, *n* = 3. **C** USP15 protein expression in AML12 cells subjected to 12 h of hypoxia followed by 0, 1, 2, 4, and 8 h of reoxygenation. **D**–**J** AAV-TBG-USP15 shRNA was injected into mice followed by liver I/R. **D** USP15 mRNA expression, *n* = 6. **E** H&E staining. Scale bar, 100 μm. **F** Serum ALT, AST and LDH levels, *n* = 8. **G** Liver IL-1β, IL-6 and TNF-α mRNA levels, *n* = 6. **H** Liver CAT, SOD, H_2_O_2_, and MDA contents, *n* = 8. **I** TUNEL staining. Scale bar, 100 μm. **J** USP15, p66Shc, Bcl2, Bax, and C-cas-3 protein expression. **K** AML12 cells were transfected with USP15 siRNA under H/R conditions. USP15, p66Shc, Bcl2, Bax and C-cas-3 protein expression, *n* = 3. ^#^*P* < 0.05, ^##^*P* < 0.01.

We further explored the role of USP15 in liver I/R. High liver expression of USP15 protein were observed in patients subjected to liver transplantation (Fig. 5A). Mice exposed to liver I/R showed a marked increase in USP15 protein, which peaked at 6 h of reperfusion (Fig. 5B). A similar trend was observed in vitro (Fig. 5C). The dramatic increase in USP15 expression in liver I/R prompted us to explore whether USP15 is involved in liver I/R. Liver-specific USP15 knockdown attenuated histological liver damage as reflected by shrinking areas of necrosis as well as decreased serum AST, ALT, and LDH levels (Fig. 5D–F). Concomitantly, USP15 silencing also restrained the liver I/R-driven inflammatory response, as measured by the levels of IL-1β, IL-6, and TNF-α, and oxidative stress response, as assessed by the levels of SOD, CAT, H_2_O_2_, and MDA (Fig. 5G, H). Considerable numbers of apoptotic cells in liver I/R were observed by TUNEL staining, and this effect was reversed by USP15 knockdown (Fig. 5I). Notably, decreased p66Shc protein in mice were observed following USP15 knockdown under I/R and basal conditions, along with the inhibition of apoptosis induced by liver I/R (Fig. 5J). Similar results were also observed in vitro (Fig. 5K). In contrast, liver-enriched USP15 exacerbated liver I/R-induced inflammation, oxidative stress, and apoptosis (Fig. S6). Collectively, USP15 plays an important role in liver I/R.

### p66Shc knockdown attenuates USP15 overexpression-potentiated apoptosis, inflammation, and oxidative stress in liver I/R

**Fig. 6:**
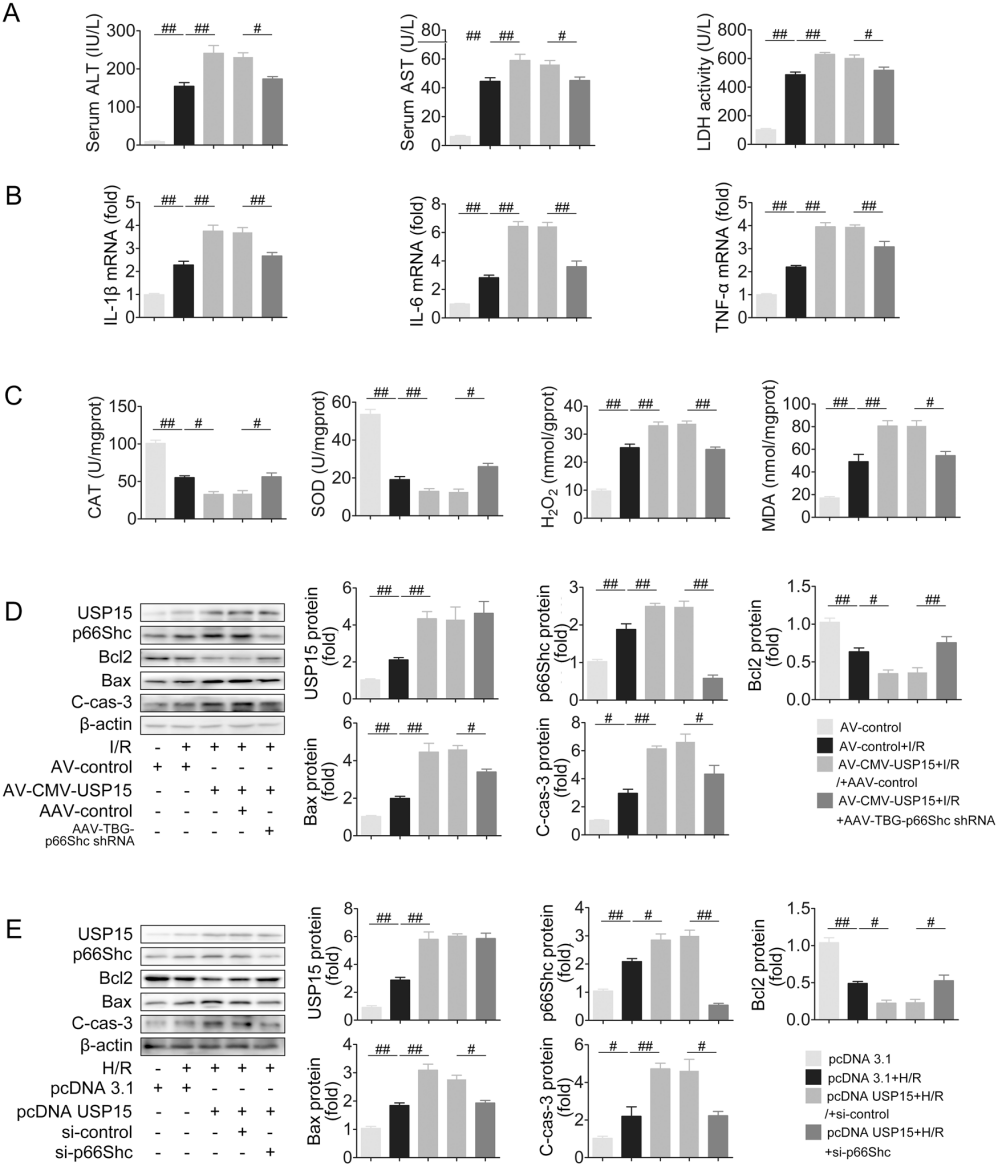
p66Shc knockdown attenuates USP15 overexpression-potentiated apoptosis, inflammation and oxidative stress in liver I/R. **A**–**D** AAV-TBG-p66Shc shRNA and AV-CMV-USP15 were successively injected to mice followed by liver I/R. **A** Serum ALT, AST and LDH levels, *n* = 8. **B** Liver IL-1β, IL-6, and TNF-α mRNA levels, *n* = 6. **C** Liver CAT, SOD, H_2_O_2_, and MDA contents, *n* = 8. **D** USP15, p66Shc, Bcl2, Bax, and C-cas-3 protein expression. **E** p66Shc siRNA and pcDNA-USP15 were co-transfected to AML12 cells followed by H/R. USP15, p66Shc, Bcl2, Bax, and C-cas-3 protein expression, *n* = 3. ^#^*P* < 0.05, ^##^*P* < 0.01.

To validate that the functional effect of USP15 in liver I/R is dependent on enhanced p66Shc expression, knockdown of p66Shc in mice was coupled with overexpression of USP15 under I/R conditions (Fig. 6 and Fig. S7). USP15 overexpression in mice increased the extent of hepatocellular necrosis (H&E staining) and liver damage (AST, ALT, LDH), and this effect was blocked by p66Shc knockdown in liver I/R (Fig. S7A and Fig. 6A). On the other hand, the increased inflammation (IL-1β, IL-6, and TNF-α) and oxidative stress (CAT, SOD, H_2_O_2_ and MDA) were markedly decreased by p66Shc silencing in liver-enriched USP15 mice (Fig. 6B, C). In support of the dependence of USP15 on p66Shc expression in liver I/R, p66Shc silencing inhibited the activation of Drp1, which protected against the detrimental effect of USP15 on mitochondrial fission and apoptosis during liver I/R (Fig. S7B and Fig. 6D). These results were confirmed in vitro. USP15-driven mitochondrial fission and apoptosis in H/R, was abrogated by p66Shc knockdown along with Drp1 inhibition (Fig. S7C, D, and Fig. 6E). In summary, p66Shc knockdown attenuates USP15 overexpression-potentiated apoptosis, inflammation and oxidative stress in liver I/R.

### p66Shc overexpression exacerbates USP15 knockdown-ameliorated apoptosis, oxidative stress and inflammation in liver I/R

**Fig. 7:**
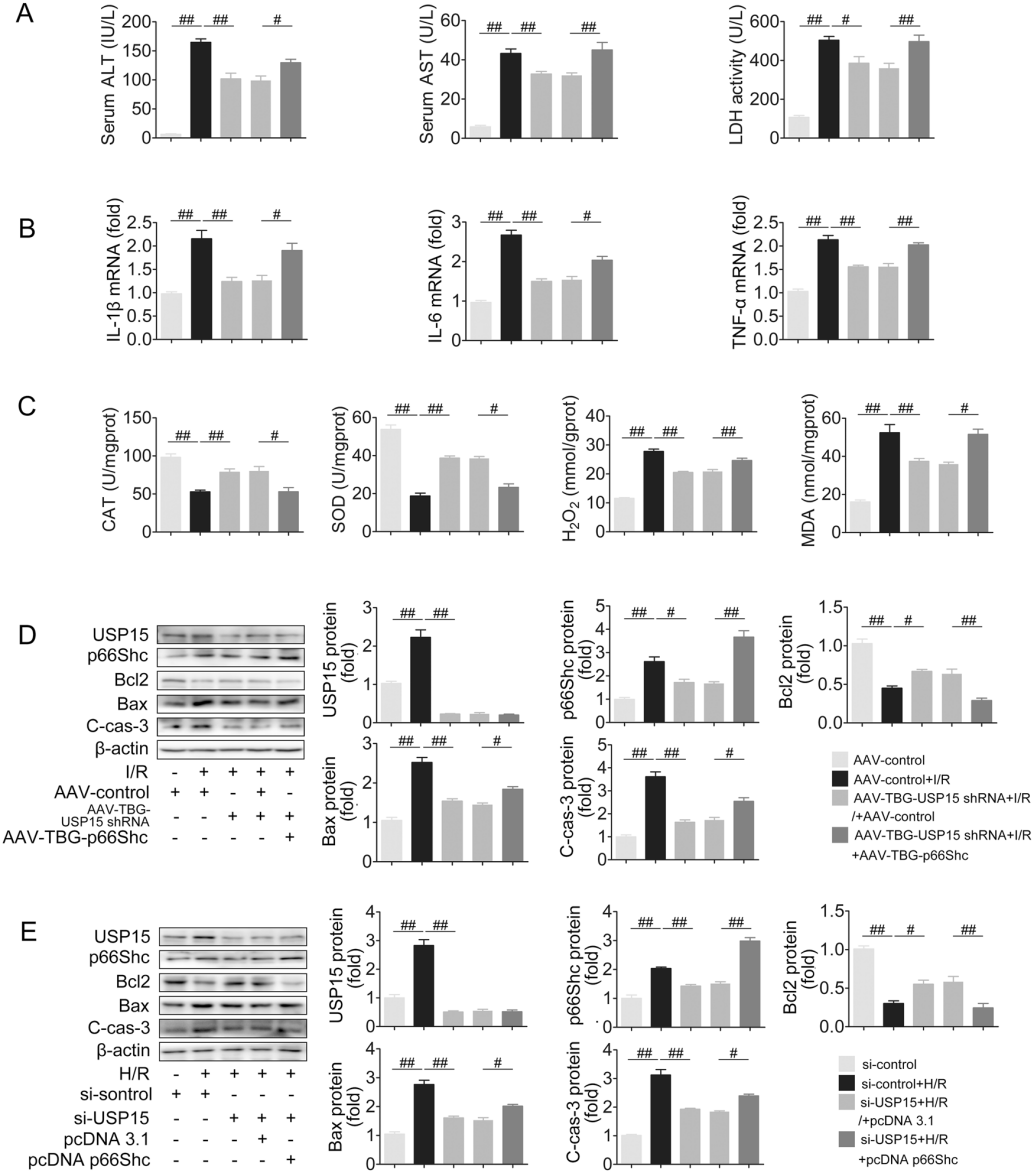
p66Shc overexpression exacerbates USP15 knockdown-ameliorated apoptosis, inflammation, and oxidative stress in liver I/R. **A**–**D** AAV-TBG-p66Shc and AAV-TBG-USP15 shRNA were successively injected to mice followed by liver I/R. **A** Serum ALT, AST and LDH levels, *n* = 8. **B** Liver IL-1β, IL-6 and TNF-α mRNA levels, *n* = 6. **C** Liver SOD, CAT, H_2_O_2_, and MDA contents, *n* = 8. **D** USP15, p66Shc, Bcl2, Bax, and C-cas-3 protein expression. **E** pcDNA-p66Shc and USP15 siRNA were co-transfected to AML12 cells followed by H/R. USP15, p66Shc, Bcl2, Bax and C-cas-3 protein expression, *n* = 3. ^#^*P* < 0.05, ^##^*P* < 0.01.

The relationship between USP15 and p66Shc was further determined by USP15 knockdown coupled with p66Shc overexpression in liver I/R conditions (Fig. 7 and Fig. S8). In vivo, liver H&E staining revealed that the smaller necrotic area caused by USP15 knockdown was disrupted by p66Shc overexpression (Fig. S8). p66Shc overexpression disturbed the decreased levels of AST, ALT, and LDH in mice with USP15 silencing (Fig. 7A). Furthermore, more inflammation and greater oxidative stress were detected upon p66Shc overexpression combined with USP15 knockdown than those resulting from only USP15 knockdown in liver I/R (Fig. 7B, C). Subsequently, p66Shc overexpression counteracted the antiapoptotic effect of USP 15 silencing (Fig. 7D). Under H/R conditions, USP15 knockdown attenuated AML12 cell apoptosis, as evidenced by increased Bcl2 levels and decreased Bax and C-cas-3 levels. These changes were abrogated by p66Shc overexpression (Fig. 7E). Thus, p66Shc overexpression exacerbates USP15 knockdown-ameliorated apoptosis, inflammation and oxidative stress in liver I/R.

## Discussion

Excessive oxidative stress is implicated in liver I/R and eventually leads to hepatocellular apoptosis [39, 40]. Adaptor protein p66Shc emerges as a sensor of oxidative stress-induced apoptosis in I/R injury [41, 42]. Intriguingly, a previous study showed that number of apoptotic cells was greatly decreased by p66Shc downregulation during liver I/R [8]. In this respect, the current study highlighted the crucial role of p66Shc in liver I/R by its knockdown or overexpression. Liver-specific knockdown of p66Shc in mice protected against liver I/R injury, along with reduced ROS production, liver inflammation and apoptosis. However, liver-specific overexpression of p66Shc produced the opposite results. Thus, p66Shc is pivotal for liver I/R injury.

Drp1 activation has recently been reported to play vital roles in I/R induced mitochondrial fission and apoptosis [13, 14]. In the present study, p66Shc silencing inhibited Drp1 activation, which attenuated the disruption in mitochondrial fission and apoptosis in liver I/R. The excessive mitochondrial fragmentation and apoptosis that were aggravated by p66Shc overexpression were abolished by Drp1 inhibition. Intriguingly, scavenging mitochondrial ROS inhibited Drp1 activation and mitochondrial fragmentation upon p66Shc overexpression, which conferred protection against I/R-induced apoptosis. Taken together, p66Shc-triggered mitochondrial fission and apoptosis are associated with Drp1 activation during liver I/R in a mitochondrial ROS-dependent manner, which provides new insights into the potential mechanism of p66Shc in liver I/R.

Although there is increasing awareness of p66Shc dysfunction, little attention has been directed to abnormal p66Shc expression. Liver I/R resulted in a substantial increase in p66Shc protein in the current study, while little change in p66Shc mRNA was observed between I/R and normal samples. We presume that ubiquitination, which is considered as the major route of protein degradation [23], may play a pivotal role in mediating p66Shc expression in liver I/R. We herein revealed that USP15 is a novel DUB that stabilizes p66Shc and blocks its degradation. Upon liver I/R injury, the elevated USP15 and p66Shc levels showed similar trends. Strikingly, the clinical relevance of USP15 and p66Shc was observed in patients subjected to liver transplantation, indicating a potential interaction between USP15 and p66Shc in liver I/R injury. p66Shc belongs to the ShcA family, which shares N-terminal phosphor-tyrosine binding domain (PTB), central a collagen homology 1 (CH1) and C-terminal Src homology 2 (SH2) domains [43]. Moreover, p66Shc contains an extra collagen homology 2 (CH2) domain in N-terminal region that confers p66Shc unique oxidative function [22]. We found that USP15 interacts with p66Shc in the SH2 domain that has been shown to be critical for protein-protein interactions [3]. Moreover, USP15 knockdown increased the ubiquitination of p66Shc, resulting in the decreased protein expression of p66Shc. The interaction between USP15 and p66Shc may be attributed to the deubiquination of p66Shc by USP15. Loss of USP15 function by USP15 catalytically inactive mutant (USP15 C269A), did not appear to be associated with p66Shc expression. Thus, USP15 interacts with p66Shc and stabilizes p66Shc expression via its deubiquitinating activity.

Existing research has highlighted the critical role of DUBs in regulating cellular proteins and the pathogenesis of apoptosis [44]. Recently evidence has indicated that USP15 is implicated in cell apoptosis. PI3K/AKT signaling is involved in USP15-mediated cell apoptosis. Furthermore, PI3K/AKT signaling can promote the translation of USP15, however, USP15 suppresses AKT signaling through a negative feedback mechanism [45, 46]. USP15 deubiquitinates pro-caspase 3, which leads to the activation of cleaved caspase-3 during paclitaxel-induced apoptosis [47]. USP15 stabilizes MDM2 and induces the apoptosis of melanoma and colon cancer cells [25]. The current study demonstrated that USP15 knockdown attenuated I/R-induced apoptosis and exerted beneficial therapeutic effects on liver I/R. The protection conferred by USP15 knockdown on liver I/R was attributed to the USP15-mediated downregulation of p66Shc and was blunted by p66Shc overexpression. In contrast, USP15 overexpression exacerbated apoptosis, inflammation, and oxidative stress in liver I/R response, while p66Shc silencing abolished these changes. Furthermore, p66Shc and USP15 were colocalized in hepatocytes, and the increased p66Shc was mainly detected in mitochondria during H/R (Fig. S5). During liver I/R, the inhibition of Drp1 activation by p66Shc silencing attenuated USP15-driven mitochondrial fission and apoptosis, which indicated that the detrimental effect of USP15 in liver I/R was associated with p66Shc-triggered Drp1 activation. Taken together, the results herein reveal a novel USP15-mediated mechanism through which hepatocellular apoptosis is achieved via maintaining p66Shc stability.

Notably, USPs play essential roles in substrate expression and represent attractive drug targets for therapies, and the clinical success of USP inhibitors has inspired the development of pharmacological interventions [48]. USP15 inhibitors, such as USP15-Inh [49] and USP15 ubiquitin variants (USP15 UbVs) [50], have been reported to improve the potency and specificity of USP15 inhibition under pathological conditions, and provide a promising therapeutic strategy based on the targeting of the USP15 catalytic domain. In light of the essential role of USP15 on p66Shc expression in the liver, specific inhibition of USP15, such as USP15 inhibitor, may represent a powerful and specific therapeutic approach for liver diseases.

In summary, p66Shc triggers mitochondrial fission and apoptosis in liver I/R associated with Drp1 activation. Disrupted p66Shc expression depends on the activity of USP15 deubiqutination. The current study sheds new light on the molecular mechanism of p66Shc and identifies USP15 as a novel mediator of p66Shc to facilitate better therapeutics for liver I/R.

## Supplementary information

Supplementary Figures

Supplementary Table 1

Supplemental materials WB

aj-checklist

## Data Availability

The datasets used and/or analyzed during the current study are available from the corresponding author on reasonable request.
