# Supplementary material for: USP15 regulates p66Shc stability associated with Drp1 activation in liver ischemia/reperfusion
Source: Cell Death Dis. 2022 Sep 26;13(9):823. doi: 10.1038/s41419-022-05277-8 (PMC9512921; doi:10.1038/s41419-022-05277-8)
Supplement: Supplementary file 1 — Supplementary Figures [file 41419_2022_5277_MOESM1_ESM.docx]

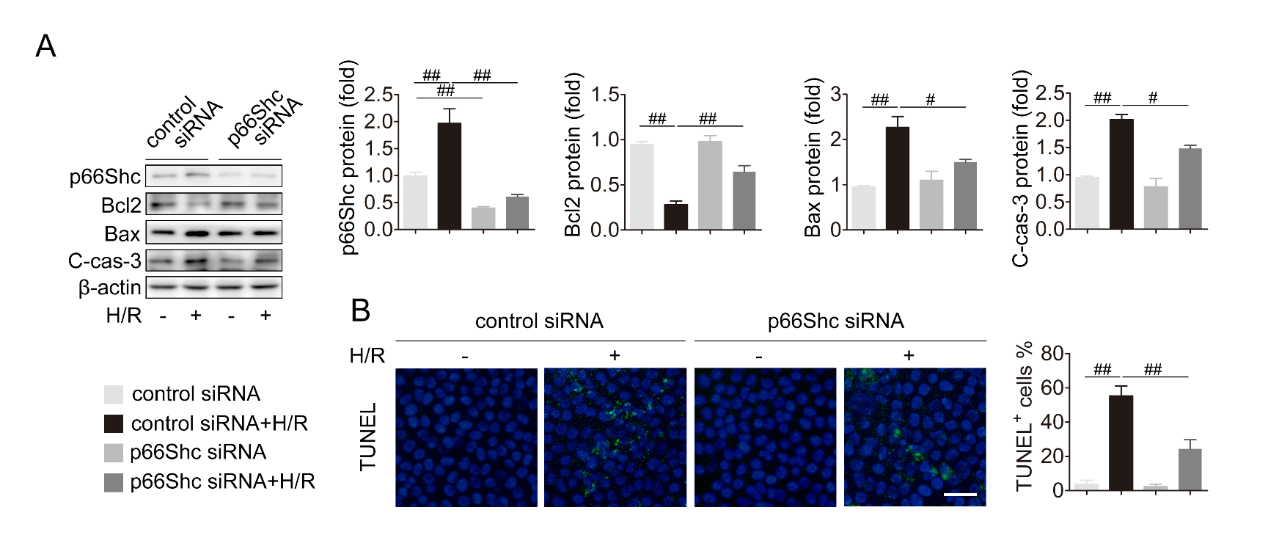


**Supplementary Figure 1. p66Shc knockdown protects AML12 cells against apoptosis under H/R conditions.** p66Shc siRNA was transfected to AML12 cells under H/R condition. (A) p66Shc, Bcl2, Bax and C-cas-3 protein expression, n=3. (B) TUNEL staining. Scale bar, 50 μm. ^#^P<0.05, ^##^P<0.01

**
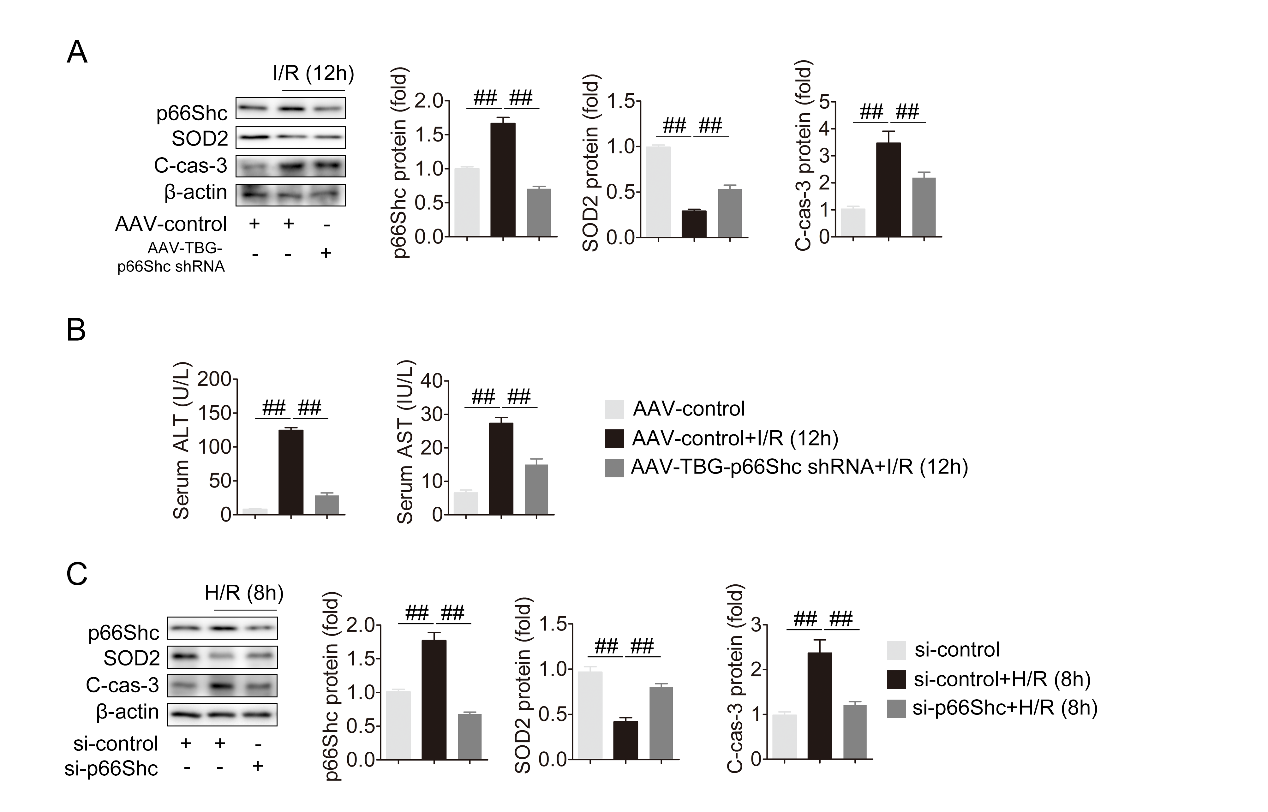
Supplementary Figure 2. p66Shc knockdown attenuates oxidative stress and apoptosis after the peak of p66Shc expression during reperfusion in liver I/R.** (A-B) AAV-TBG-p66Shc shRNA was injected into mice followed by liver I/R. After 1 h of ischemia followed by 12h of reperfusion, p66Shc, SOD2, C-cas-3 protein and serum ALT and AST were detected. (C) p66Shc siRNA was transfected to AML12 cells under H/R condition. After 12 h of hypoxia followed by 8 h of reoxygenation, p66Shc, SOD2, C-cas-3 protein were detected. ^#^P<0.05, ^##^P<0.01.

**
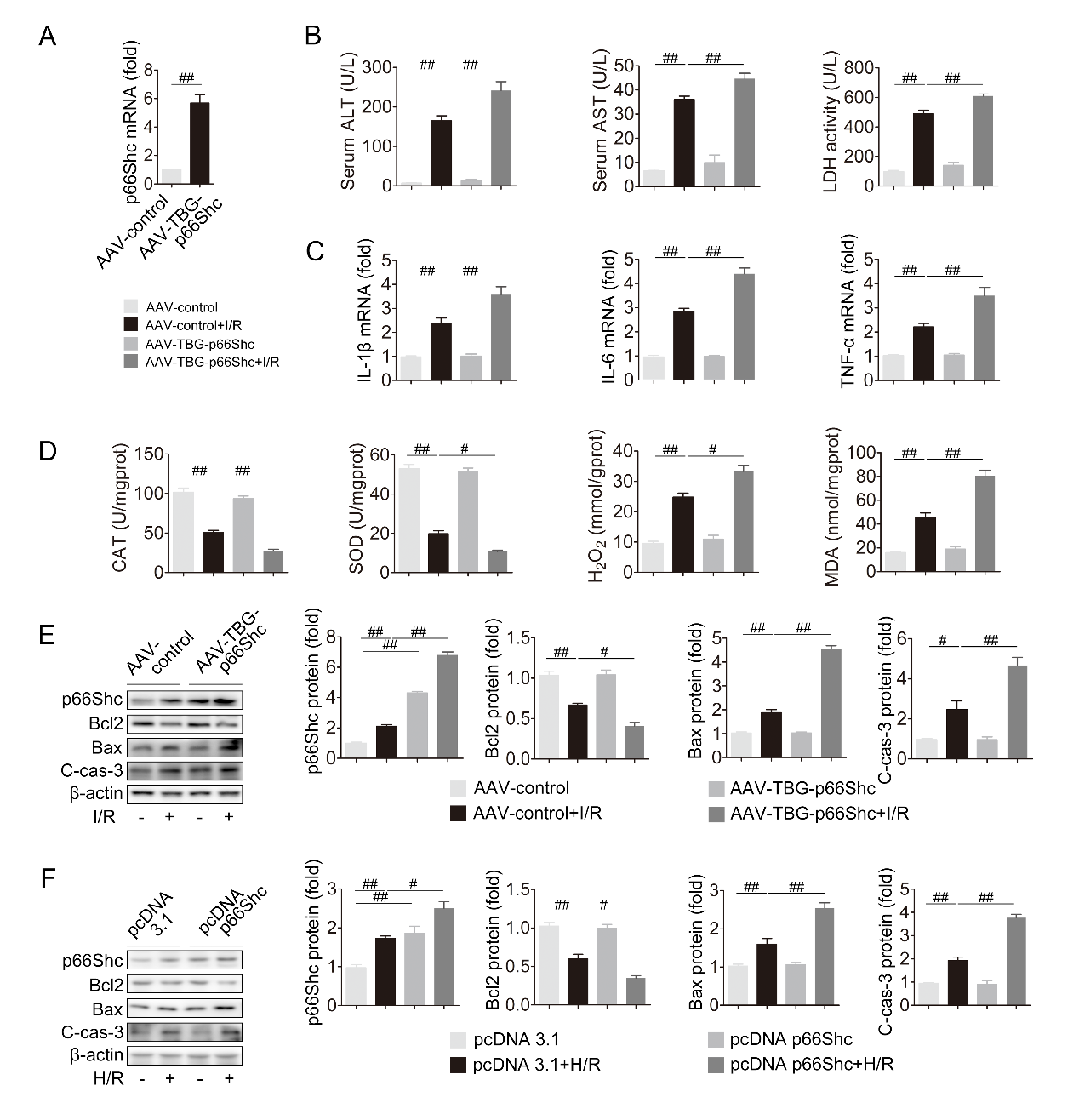
Supplementary Figure 3. p66Shc overexpression aggravates apoptosis, inflammation and oxidative stress in liver I/R.** (A-E) AAV-TBG-p66Shc was injected into mice followed by liver I/R. (A) p66Shc mRNA expression, n=6. (B) Serum ALT, AST and LDH levels, n=8. (C) Liver IL-1β, IL-6 and TNF-α mRNA levels, n=6. (D) Liver CAT, SOD, H_2_O_2_ and MDA contents, n=8. (E) p66Shc, Bcl2, Bax and C-cas-3 protein expression. (F) pcDNA-p66Shc was transfected into AML12 cells under H/R conditions. p66Shc, Bcl2, Bax and C-cas-3 protein expression, n=3. ^#^P<0.05, ^##^P<0.01.

**
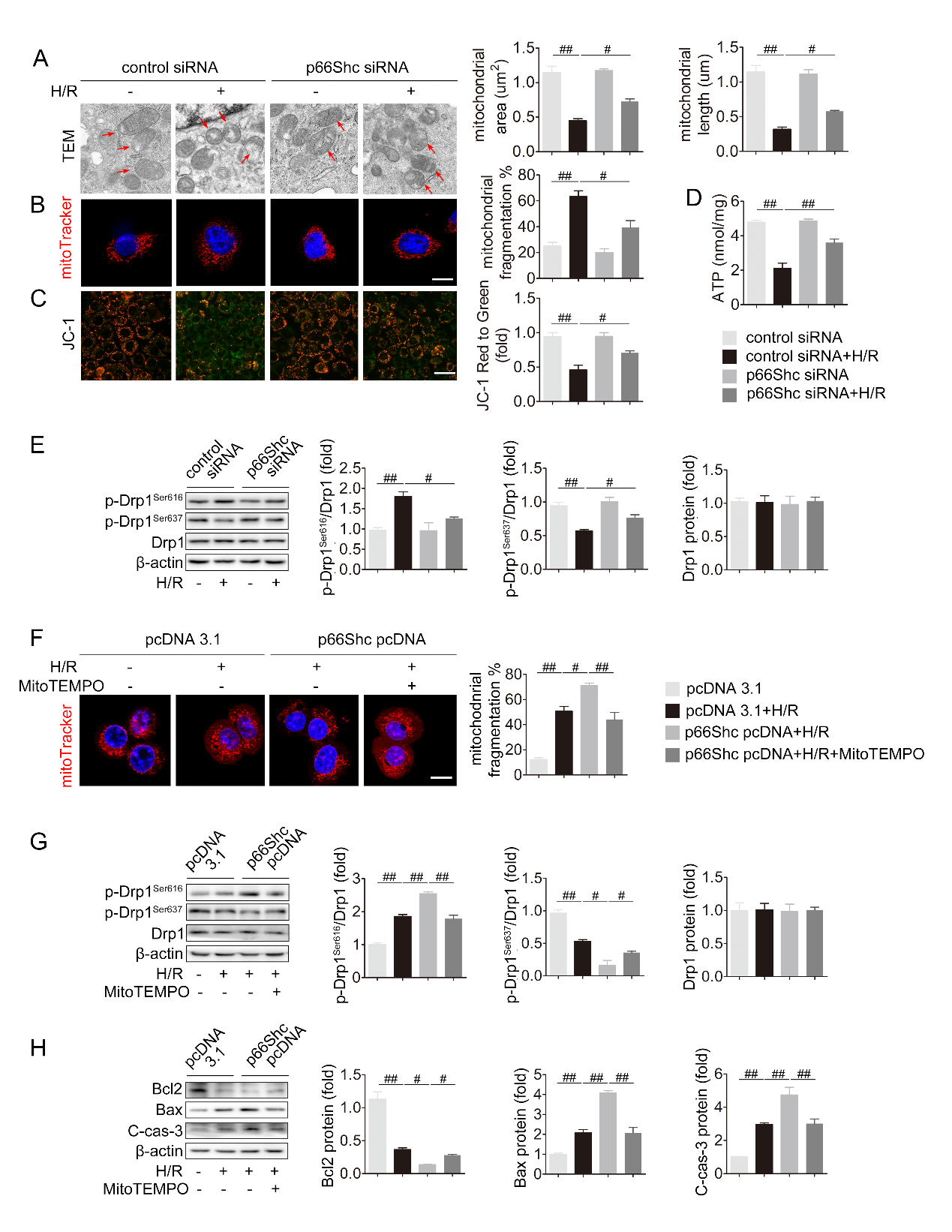
Supplementary Figure 4. p66Shc-mediated mitochondrial fission and apoptosis during liver I/R is attributed to mitochondrial ROS overproduction.** (A-E) p66Shc siRNA was transfected into AML12 cells under H/R condition. (A) Mitochondrial morphology measured using TEM (12000×, magnification). Red arrows indicate mitochondria. (B) Mitochondrial fragmentation quantified by MitoTracker staining. Scale bar, 12.5 μm. (C) JC-1 staining. Scale bar, 50 μm. (D) ATP contents, n=6. (E) p-Drp1^Ser616^, p-Drp1^Ser^^637^ and Drp1 protein expression. (F-H) AML12 cells were transfected with pcDNA-p66Shc and then stimulated with MitoTEMPO in response to H/R. (F) Mitochondrial fragmentation quantified by MitoTracker staining. Scale bar, 12.5 μm. (G) p-Drp1^Ser616^, p-Drp1^Ser637^ and Drp1 protein. (H) Bcl2, Bax and C-cas-3 protein expression, n=3. ^#^P<0.05, ^##^P<0.01.

~~
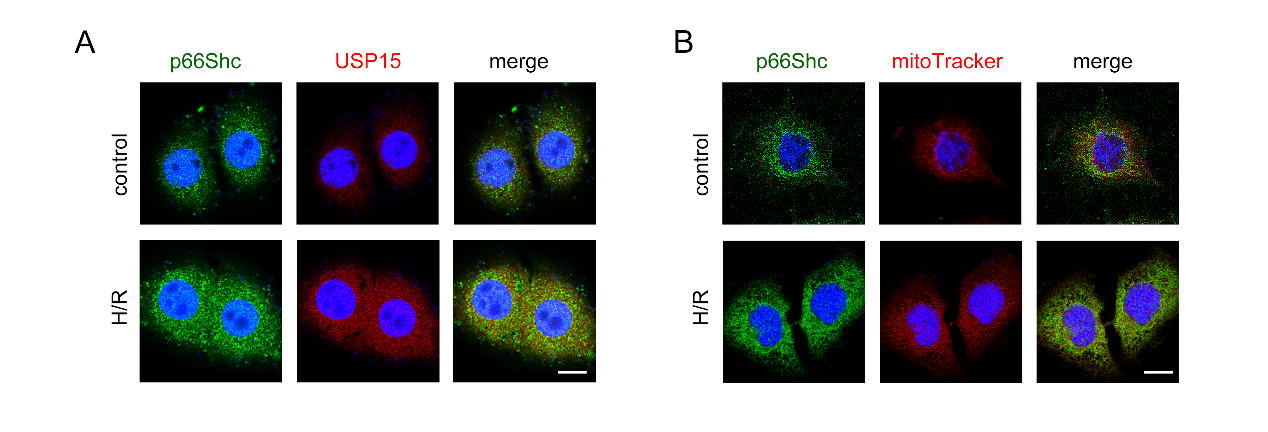
~~

**Supplementary Figure 5. p66Shc and USP15 were colocalized in hepatocytes during H/R.** AML12 cells were exposed to H/R. (A) Dual immunofluorescence staining for p66Shc (green) and USP15 (red). (B) The colocalization of p66Shc (green) and mitochondria (Mito-Tracker staining, red), Scale bar, 12.5 μm.

**
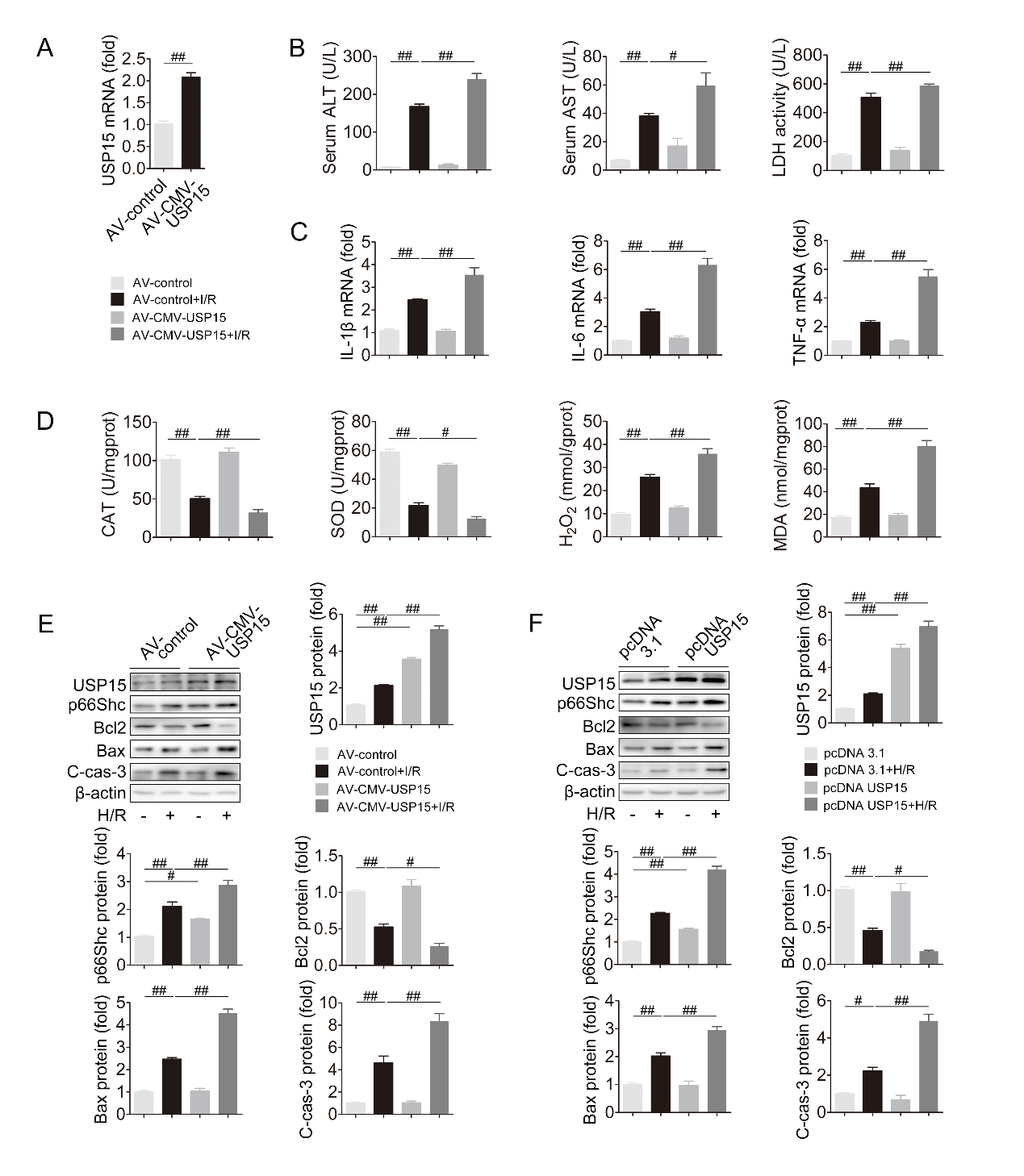
Supplementary Figure 6. USP15 overexpression exacerbates apoptosis, inflammation and oxidative stress in liver I/R.** (A-E) AV-CMV-USP15 was injected into mice followed by liver I/R. (A) USP15 mRNA expression, n=6. (B) Serum ALT, AST and LDH levels, n=8. (C) Liver IL-1β, IL-6 and TNF-α mRNA levels, n=6. (D) Liver CAT, SOD, H_2_O_2_ and MDA contents, n=8. (E) USP15, p66Shc, Bcl2, Bax and C-cas-3 protein expression. (F) pcDNA-USP15 was transfected into AML12 cells under H/R conditions. USP15, p66Shc, Bcl2, Bax and C-cas-3 protein expression, n=3. ^#^P<0.05, ^##^P<0.01.


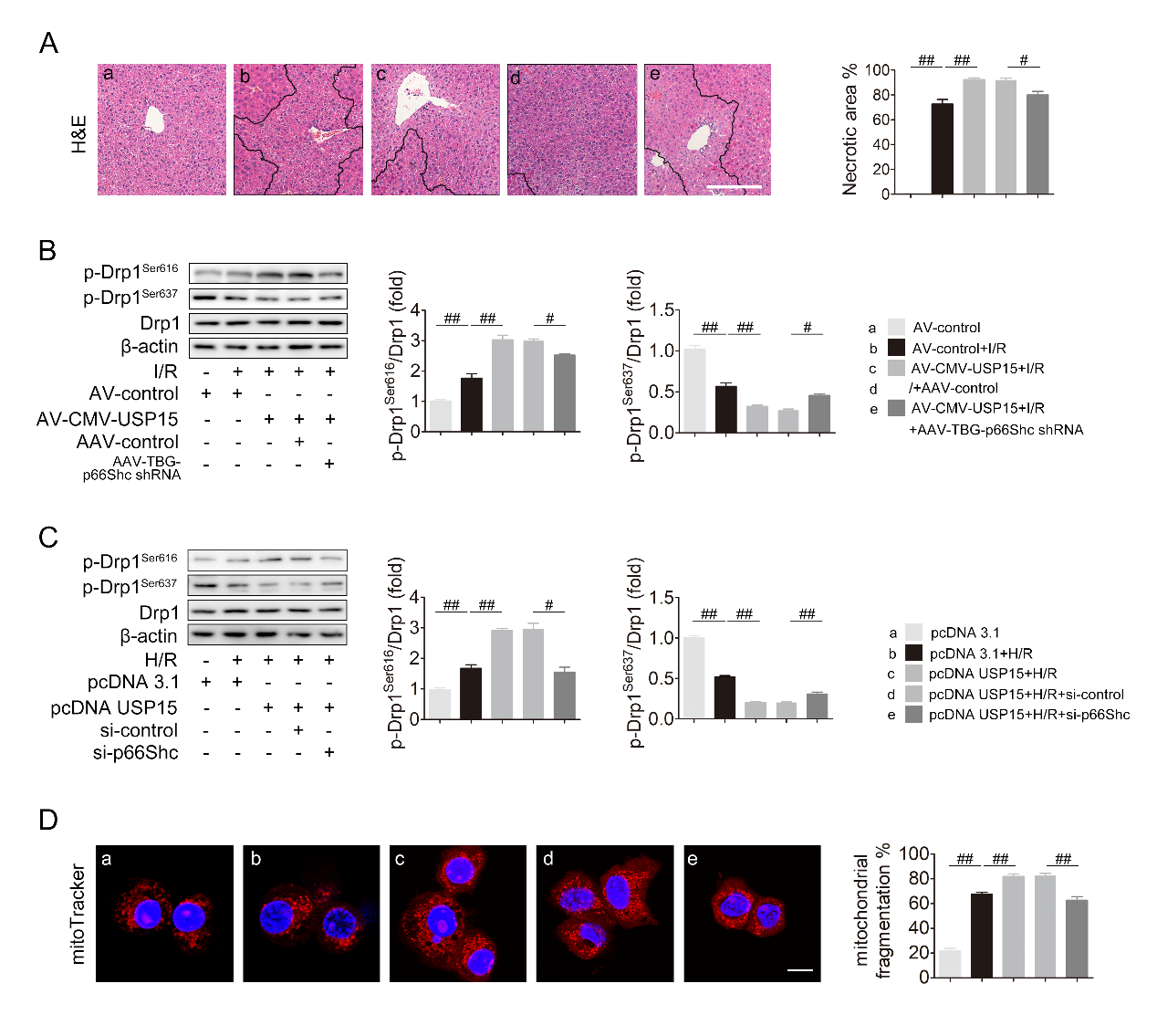


**Supplementary Figure 7. p66Shc knockdown attenuates USP15 overexpression-potentiated histological injury and mitochondrial fission in liver I/R.** (A, B) AAV-TBG-p66Shc shRNA and AV-CMV-USP15 were successively injected to mice followed by liver I/R. (A) H&E staining. Scale bar, 100 μm. (B) p-Drp1^Ser616^, p-Drp1^Ser637^ and Drp1 protein expression. (C, D) p66Shc siRNA and pcDNA-USP15 were co-transfected to AML12 cells followed by H/R. (C) p-Drp1^Ser616^, p-Drp1^Ser637^ and Drp1 protein expression, n=3. ^#^P<0.05, ^##^P<0.01. (D) Mitochondrial fragmentation quantified by MitoTracker staining. Scale bar, 12.5 μm.


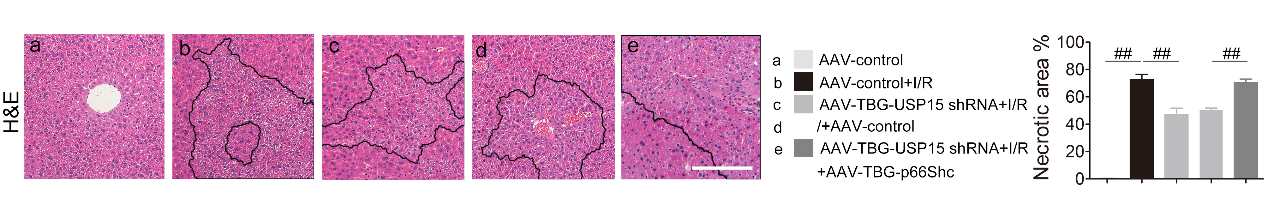


**Supplementary Figure 8. p66Shc overexpression exacerbates USP15 knockdown-ameliorated histological injury in liver I/R.** AAV-TBG-p66Shc and AAV-TBG-USP15 shRNA were successively injected to mice followed by liver I/R. H&E staining. Scale bar, 100 μm.
