# Supplementary material for: USP15 regulates p66Shc stability associated with Drp1 activation in liver ischemia/reperfusion
Source: Cell Death Dis. 2022 Sep 26;13(9):823. doi: 10.1038/s41419-022-05277-8 (PMC9512921; doi:10.1038/s41419-022-05277-8)
Supplement: Supplementary file 2 — Supplementary Table 1 [file 41419_2022_5277_MOESM2_ESM.docx]

**Supplementary Table 1. Information of clinical samples**

|  | Control | Liver transplantation |
| --- | --- | --- |
| Age (years) | 40.5±10.6 | 44.3±9.3 |
| Gender (M/F) | 5/1 | 4/2 |
| Time of biopsy | 2019.5.1-2020.12.20 | |
| Medical history | All donors were not diagnosed with hepatitis and steatosis | |
| Donation Status | Donation after brain death | |
