# Supplementary material for: USP15 regulates p66Shc stability associated with Drp1 activation in liver ischemia/reperfusion
Source: Cell Death Dis. 2022 Sep 26;13(9):823. doi: 10.1038/s41419-022-05277-8 (PMC9512921; doi:10.1038/s41419-022-05277-8)

Figure 1A

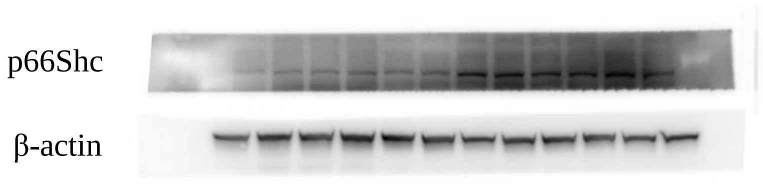

Figure 1B

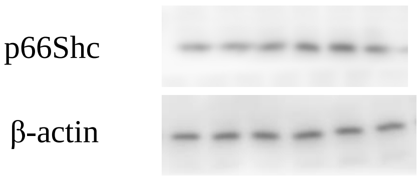

Figure 1J

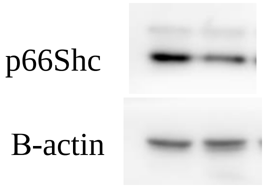

Figure 2E

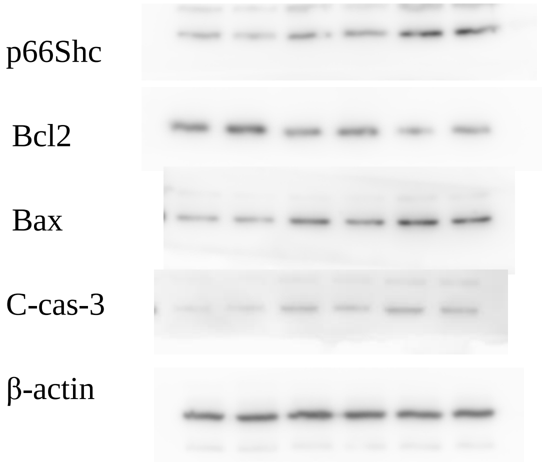

Figure 1J

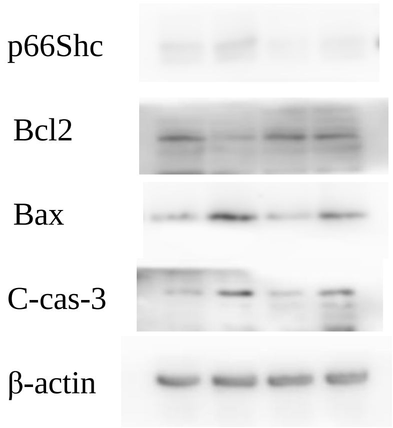

Figure 3B

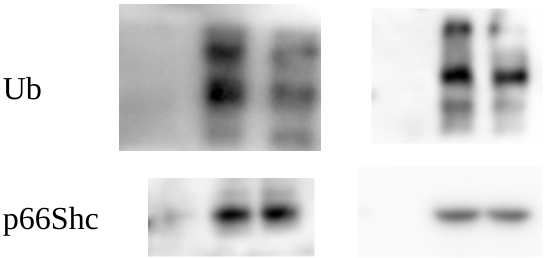

Figure 2B

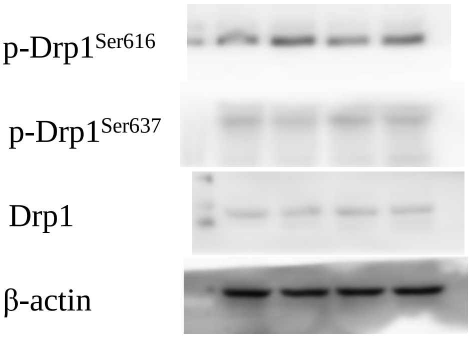

Figure 3D

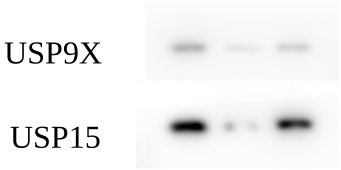

Figure 3F

IB: USP15

IB: p66Shc

Input: USP15

Input: p66Shc

Input: β-actin

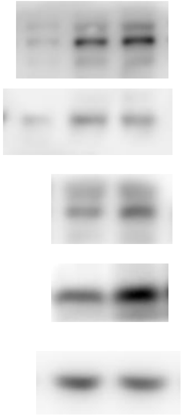

IB: p66Shc

IB: USP15

Input: USP15

Input: p66Shc

Input: β-actin

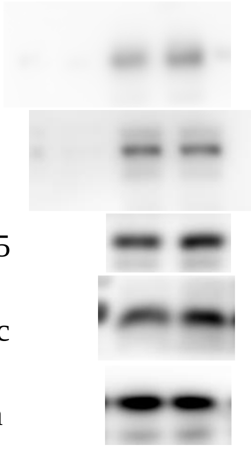

Figure 3G

IB: USP15

IB: p66Shc

Input: USP15

Input: p66Shc

Input: β-actin

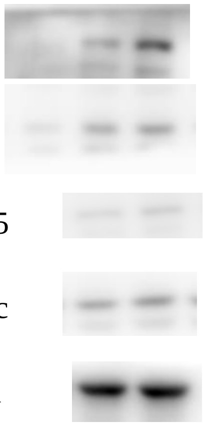

IB: p66Shc

IB: USP15

Input: USP15

Input: p66Shc

Input: β-actin

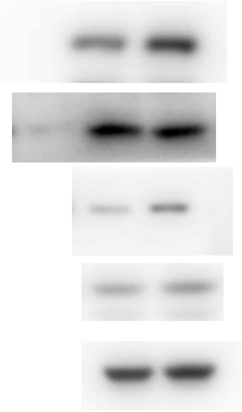

Figure 3H

IP: Myc IB: Flag

WCL IB: Flag

WCL IB: Myc

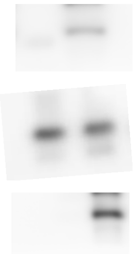

IP: Flag IB: Myc

WCL IB: Myc

WCL IB: Flag

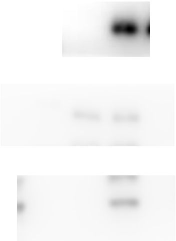

Figure 3J

IP: Myc IB: Flag

WCL IB: Flag

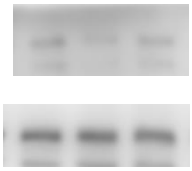

Figure 4A

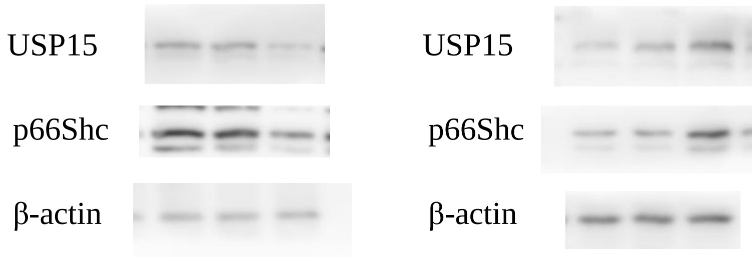

Figure 4C

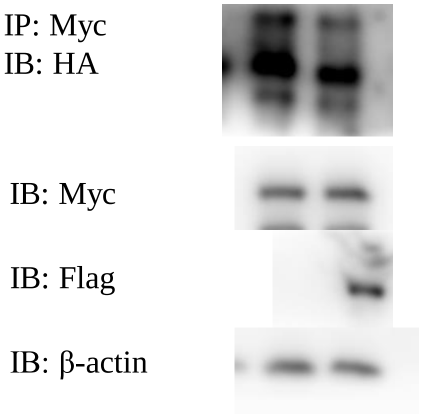

Figure 4D

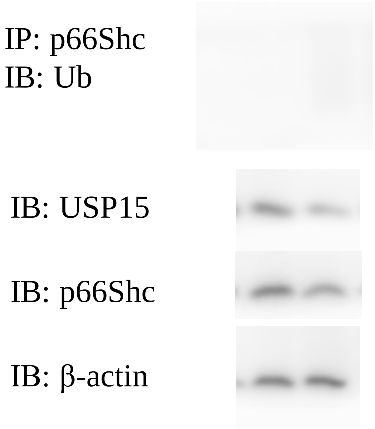

Figure 4E

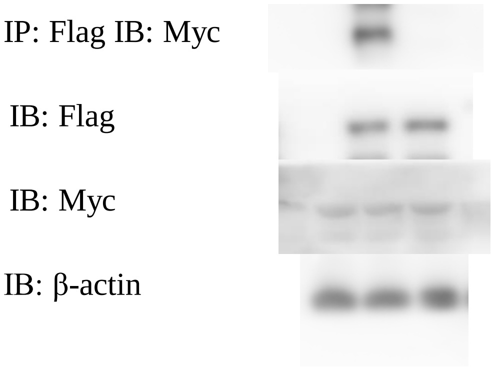

Figure 4F

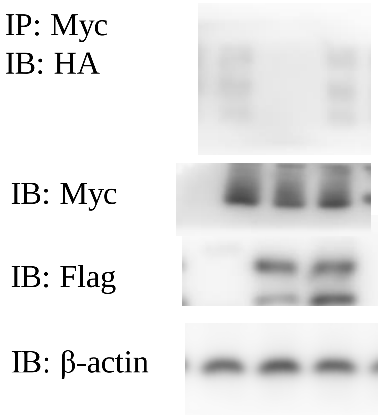

Figure 4G

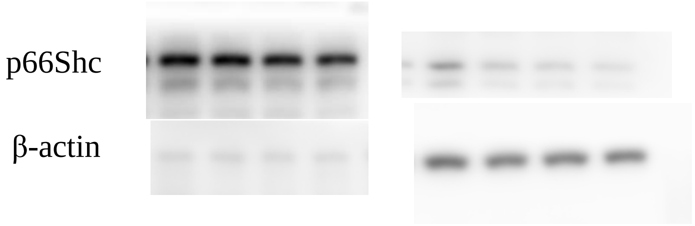

Figure 4H

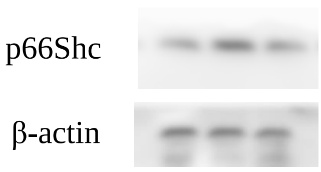

Figure 4I

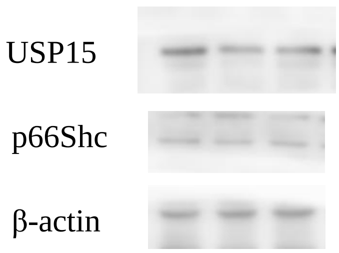

Figure 5A

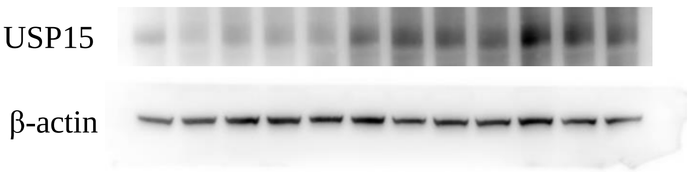

Figure 5B

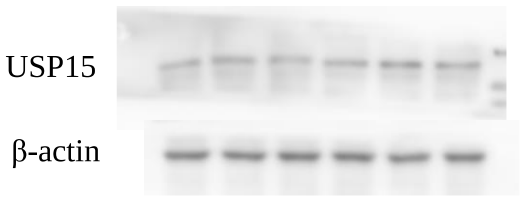

Figure 5C

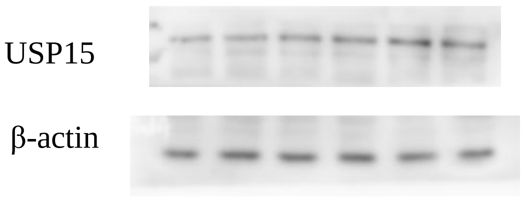

Figure 5J

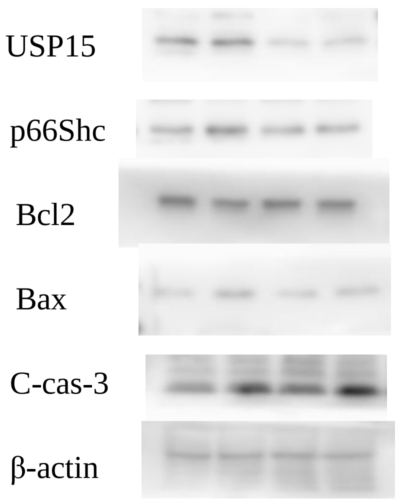

Figure 5K

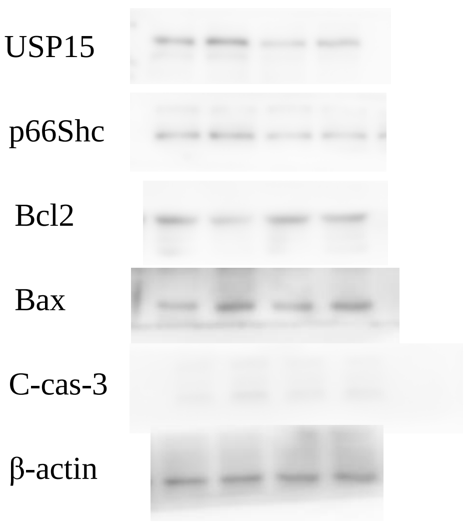

Figure 6D

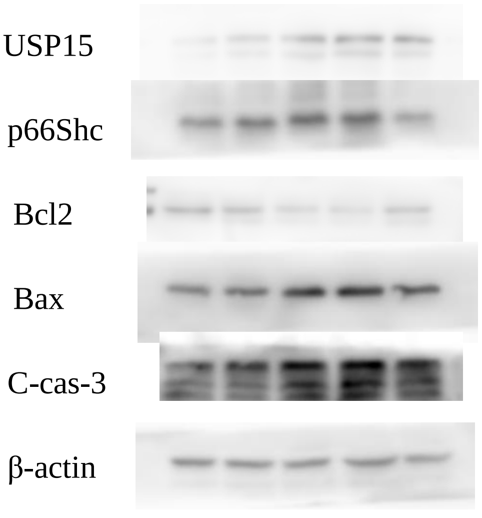

Figure 6E

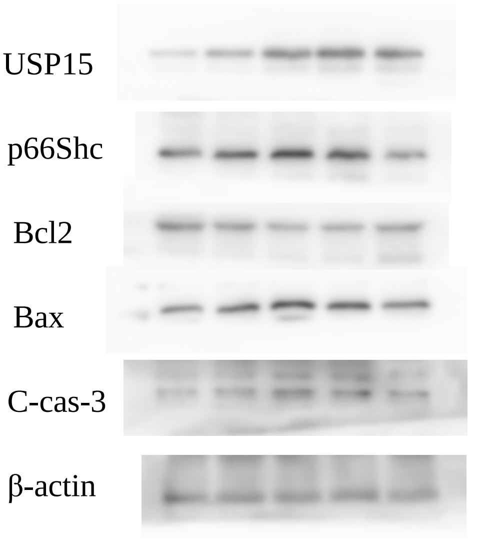

Figure 7D

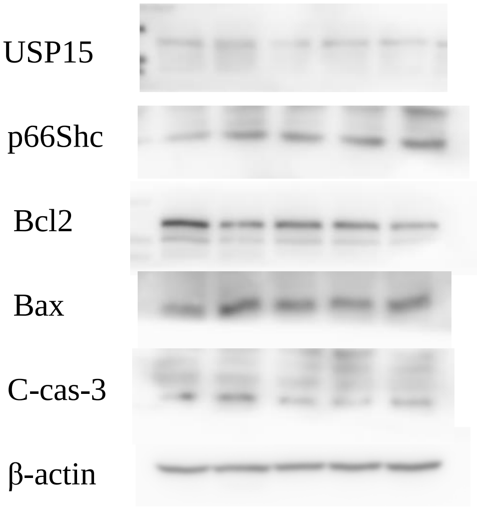

Figure 7E

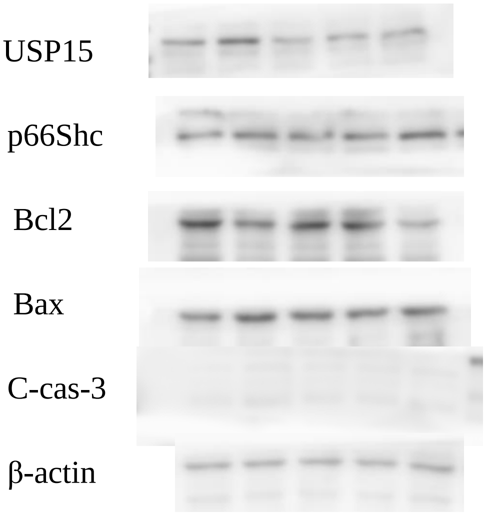

Supplementary Figure 1A

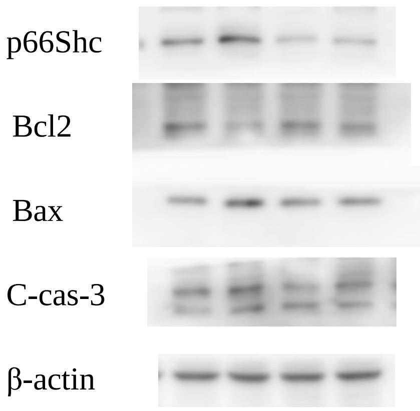

Supplementary Figure 2A, 2C

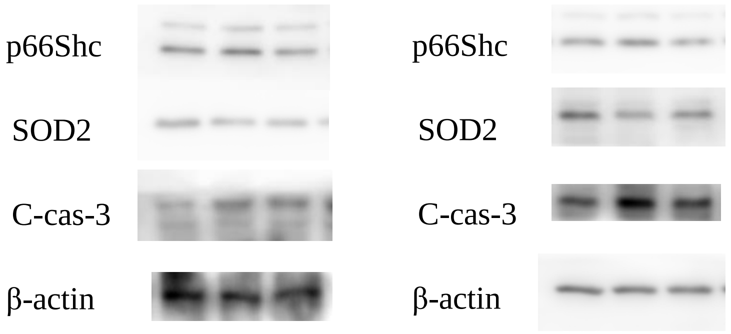

Supplementary Figure 3E

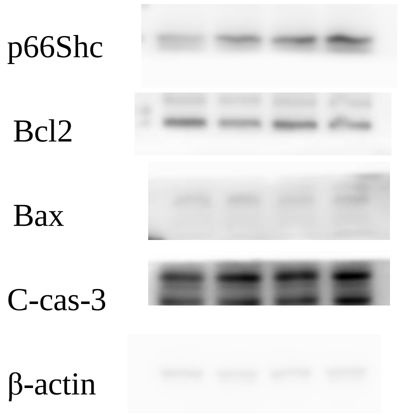

Supplementary Figure 3F

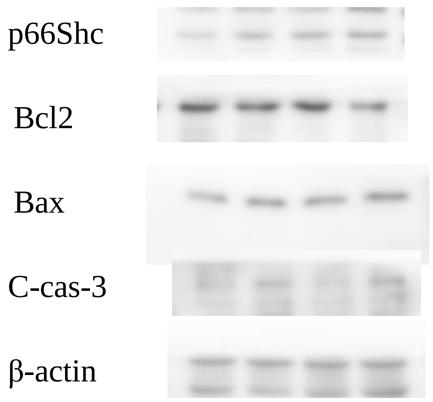

Supplementary Figure 4E

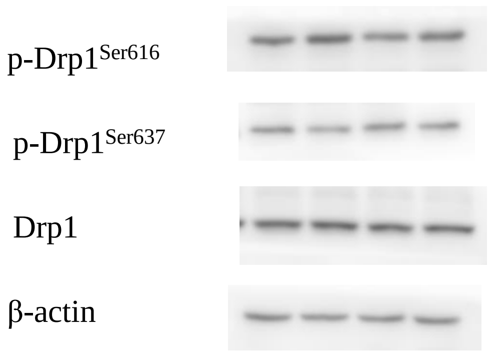

Supplementary Figure 4G

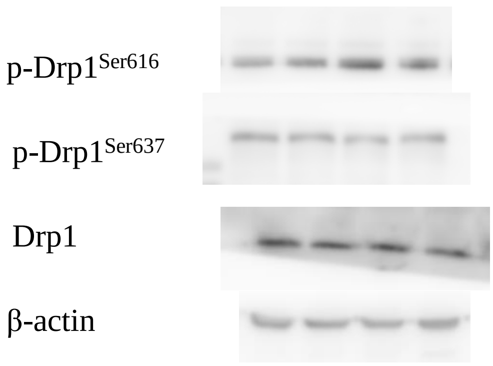

Supplementary Figure 4H

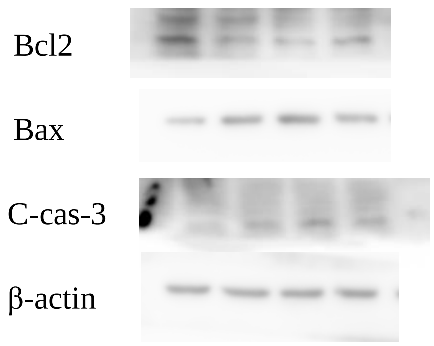

Supplementary Figure 6E

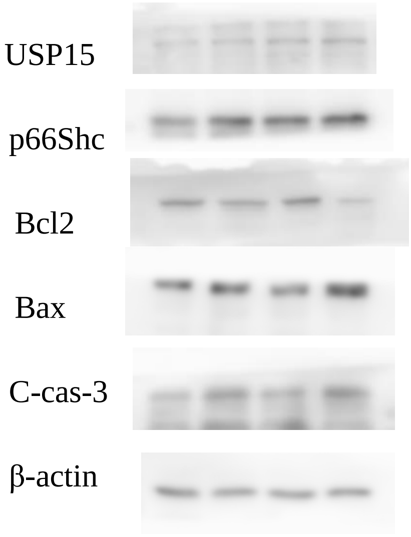

Supplementary Figure 6F

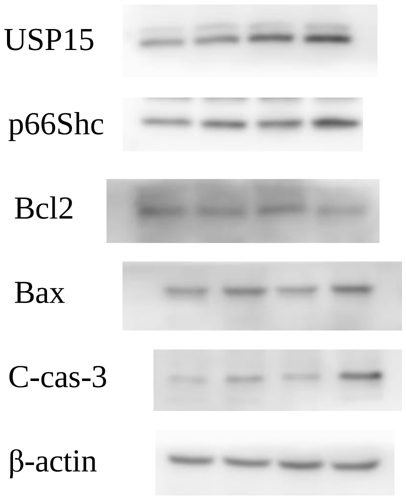

Supplementary Figure 7B

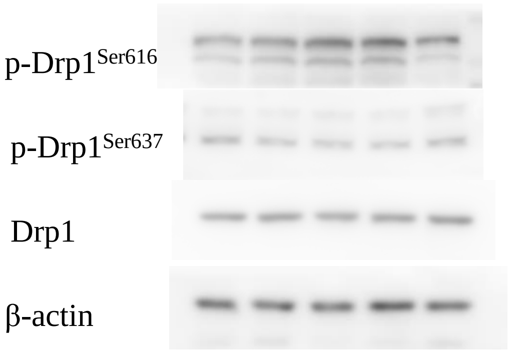

Supplementary Figure 7C

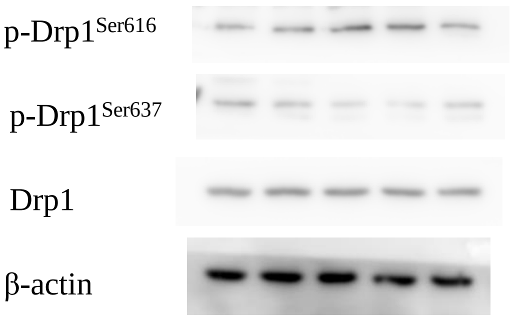

Supplement: Supplementary file 3 — Supplemental materials WB [file 41419_2022_5277_MOESM3_ESM.pdf]
